# The systematic techno-stylistic and chemical study of glass beads from post-15th century West African sites

**DOI:** 10.1371/journal.pone.0318588

**Published:** 2025-02-10

**Authors:** Miriam Truffa Giachet, Bernard Gratuze, Denis Genequand, Yao Serge Bonaventure Loukou, Éric Huysecom, Anne Mayor

**Affiliations:** 1 Laboratoire Archéologie Africaine & Anthropologie (ARCAN), Faculty of Science, University of Geneva, Geneva, Switzerland; 2 Centre Ernest-Babelon de l’Institut de Recherche sur les Archéomatériaux (IRAMAT-CEB, UMR 7065), CNRS/University of Orléans, Orléans, France; 3 Site et Musée romains d’Avenches, Avenches, Switzerland; 4 History Department, Faculty of Arts and Humanities, University Cheikh Anta Diop of Dakar, Dakar, Senegal; 5 Global Studies Institute, University of Geneva, Geneva, Switzerland; University of Michigan, UNITED STATES OF AMERICA

## Abstract

The systematic chemical analysis of large collections of archaeological glass beads is essential to better understand trade patterns at different times around the world. Glass beads’ trade towards and within sub-Saharan West Africa grew exponentially over time to culminate with the establishment of the Atlantic Trade. Although these artefacts are very commonly found in archaeological contexts dating after the 15^th^ century CE, the assemblages are generally poorly studied from a chemical point of view. We present here the study of 916 glass beads found in five archaeological sites in Ghana, Mali, and Senegal, in contexts dated between the 15^th^ and the mid-20^th^ century CE. Besides the techno-stylistic classification of the whole assemblage, the compositional study of a sub-group of 578 monochrome and polychrome glass beads was performed. The 798 glass samples composing the selected beads were therefore classified based on their main chemical composition. Moreover, major, minor, and trace elements analysis by Laser Ablation-Inductively Coupled Plasma-Mass Spectrometry (LA-ICP-MS) and the statistical analysis of the results by Principal Component Analysis (PCA) led to the identification of the probable origin of the glass. Different suppliers were distinguished for the Ghanaian earlier beads and the Senegalese and Malian later ones, in relation to the different European trade partners at different times.

## Introduction

### Scope and objectives

Glass beads are a very important class of archaeological artefacts as their typological and chemical study can help shed a light on the glass production technologies and trade patterns of a specific region. In sub-Saharan West Africa, glass beads have mainly been prestige goods that arrived in this part of the continent via trans-Saharan or oceanic trade depending on the period, or were produced locally from imported glass. The provenance of the beads, and consequently the commercial flows, are more and more well-defined for the ancient and medieval trans-Saharan trade also because of the increasing chemical characterization of archaeological assemblages. Conversely, for more recent periods, the analysis of the dynamics of exchange is mainly based on historical sources, generally ascribing as “European” the source of glass artefacts. This is due to the lack of systematic chemical studies of large archaeological and historical glass beads assemblages, which would be fundamental in view of refining the provenance studies of these traded goods.

We present here the techno-stylistic and chemical characterisation of 916 glass beads found in five archaeological sites in Senegal, Mali, and Ghana in the framework of the research project “Human Population and Palaeoenvironment in Africa (HPPA)” coordinated by the laboratory Archaeology and Peopling of Africa (APA), today ARChaeology of Africa & ANthropology (ARCAN), at the University of Geneva in collaboration with several research groups in Switzerland, France, Germany, Mali, Senegal, and Ghana [1].

### Glass beads trade in West Africa

The first glass beads reached sub-Saharan West Africa in the first millennium BCE through sporadic exchanges across the Sahara Desert [2, 3]. From the 8^th^ century CE, trans-Saharan trade routes progressively developed to peak between the 10^th^ and the 15^th^ century CE with the expansion of the Sahelian states. Among other commodities, glass beads were exchanged at different spatial scales, from the large trade centres to the rural areas [4]. Trans-Saharan trade then gave way to the Atlantic trade with the Europeans and the Americans from the 15^th^ century onward, although it never completely disappeared. Outposts were built along the coasts of West Africa first by the Portuguese between the 16^th^ and the 17^th^ century, then by the Dutch, the French, and the British between the 17^th^ and the 19^th^ century [5, 6]. The main good sought by the Europeans at the very beginning was gold, but then ivory, pepper, precious wood, leather, and slaves became valuable trade goods, exchanged for textiles, guns, metals, alcohol, and glass produced in Europe. Historical documents such as travel accounts, invoices, and detailed trade reports, as well as archaeological finds, like in Elmina, Ghana [7–9], in Garumele, Niger [10], and in the Falémé Valley and other sites in Senegal [11–13], show how glass beads were especially important commodities, in large demand in West Africa [7, 12, 14–17].

Glass beads traded by Europeans in West Africa had specific typologies depending on the good they were exchanged for, and sample cards and catalogues were created by the beadmakers to categorize the items [18–21]. Distinctive qualifying terminology was hence used based on the origin and the type of the beads, as well as depending on the recipients of the goods [16: 22].

Local glass beads production has also been attested in various West African sites where imported glass items were reprocessed to create beads according to local taste [22–25].

### Glass production in the Mediterranean basin and Europe

The ingredients to produce man-made glass have changed at different historical times depending on raw material availability, technological knowledge, and market demand. The chemical characterization of archaeological glass artefacts and production waste showed that the main ingredients used for glass manufacture in the Mediterranean basin and Middle East between the 2^nd^ millennium and the 9^th^ century BCE were silica and halophyte plant ash [26–28], to switch to quartz limestone sand and mineral flux (mainly *natron*) between the 10^th^ century BCE and the 9^th^ century CE [29–32], and to go back again to vegetal flux (plant ash) from the 8^th^ century CE onwards [33–35], although in the Middle East, east of the Euphrates River, the use of plant ash probably never ceased and resumed on a larger scale around the 3^rd^ century CE [36–38]. Around the same time, in continental Europe glass artisans started to use wood ash as flux to lower the temperature of quartz sand and lime as glass stabilizer, leading to the production of potassic and, later, mixed akali glass. This transition was progressive, and many different recipes were ventured from the 8^th^ to the 16^th^ century CE, showing a gradual increase in CaO and P_2_O_5_ content and a progressive decrease in K_2_O content [27, 39–41]. Besides, the use of quartz sand, beech ash, and potash led to the production of the Bohemian glass in the regions of Bohemia and Silesia [40]. Another important production in Medieval Europe was lead glass. The use of lead to lower the glass working temperature was sporadic in the British Isles around the 10^th^–11^th^ century CE and in Eastern Europe in the 12^th^–13^th^ century CE, and more intense in Central and Northern Europe from the 16^th^ century CE onwards [27, 42]. Finally, one of the most important glass productions in medieval and post-medieval Europe was the Italian glass manufacture (mainly in Liguria, Tuscany, and Veneto [43–46]). The production of soda-lime glass in Northern Italy started in the 6^th^–7^th^ century CE with the importation and reworking of raw glass produced in the Middle East, making the distinction between the two types very difficult. It is not before the 13^th^ century CE that primary production of glass in Murano and Venice started, using plant ash imported first from the Middle East and from Spain, and Southern France later, as well as the silica source imported from Ticino, in Switzerland [46]. The Venetian glass industry flourished and led the glass market between the 13^th^ and the 17^th^ century CE, showing a constantly developing technology, an increasing glass quality (from common glass to *Vitrum Blanchum* to *Cristallo*), and a vast variety of artefact types produced. Around the 16^th^ century the raw materials supply became scarce, and glass artisans began to move towards Central and Northwestern Europe, taking with them the secrets of the Venetian glassmaking [46, 47]. Flawless imitations of the prestigious Venetian glass, both from a typological and a chemical point of view (the so-called *Façon de Venise*), started to sprout especially in the Netherlands, in British islands, and in France, effectively putting an end to the Venetian glassmaking monopoly at the end of the 17^th^ century. Venice remained, however, an active and dynamic player in the glass beads market until the 19^th^ century [17]. In the 19^th^ century new industrial materials were introduced in the European glassmaking process, such as synthetic soda and potash for the base glass, fluorine as an opacifier, as well as various chromophores like nickel for grey glass, uranium for yellow glass, and cadmium and selenium for red glass [48, 49].

## Materials and methods

Permission for the temporary exportation and study of the glass beads was granted by the ISH (Institut des Sciences Humaines) for Mali, IFAN (Institut Fondamental d’Afrique Noire) for Senegal, and GMMB (Ghana Museums and Monuments Board) for Ghana.

### Archaeological contexts and glass bead assemblages

#### Old Buipe, Ghana

**Fig 1:**
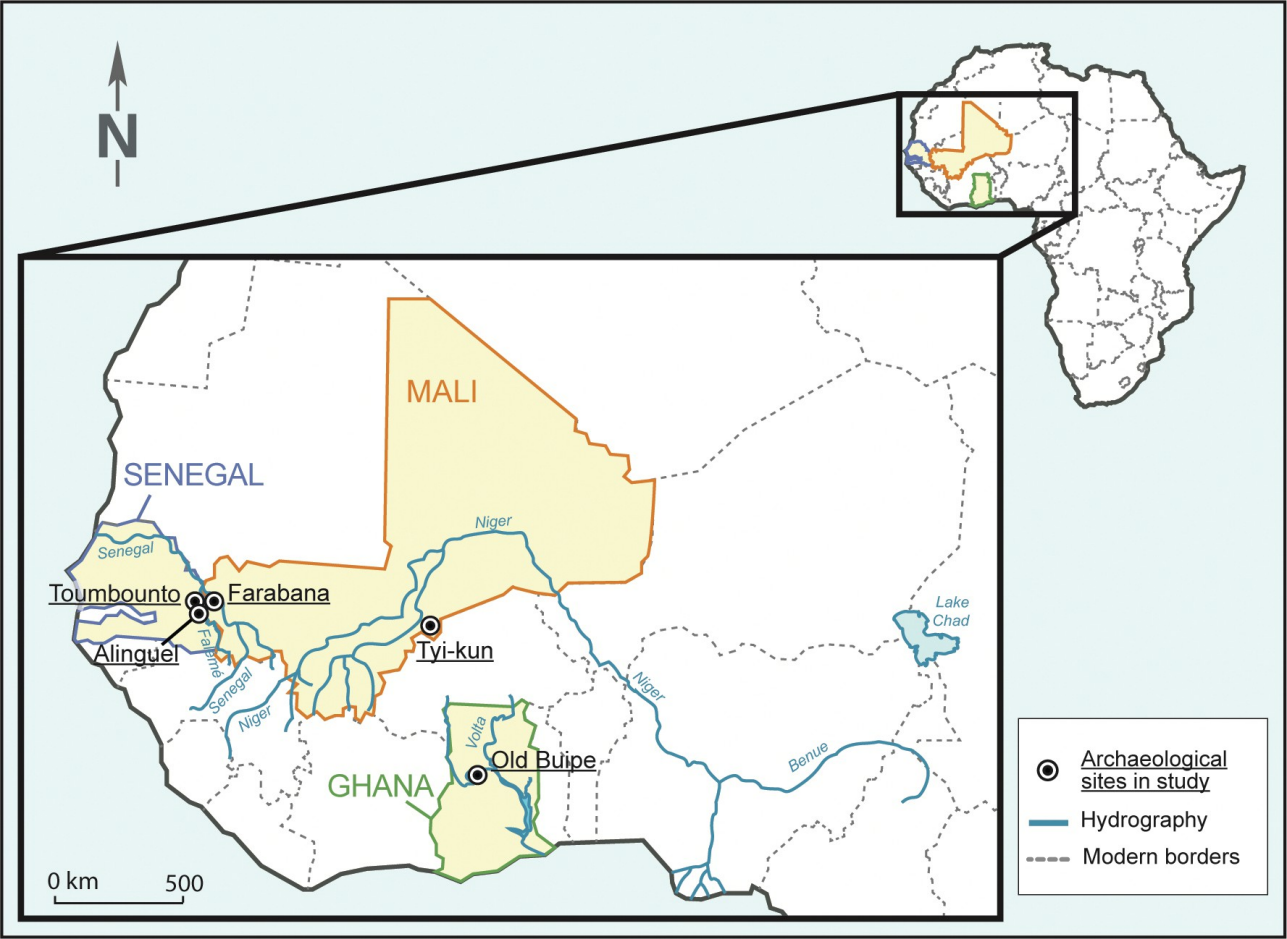
Location of the archaeological sites in West Africa. The West African archaeological sites where the analysed glass beads were found (map: courtesy of CIA’s The World Factbook 2020, modified).

Old Buipe site is located 10 km north of the Black Volta River, close to its confluence with the White Volta River, in north-central Ghana (Fig 1).

According to written sources and oral traditions, Old Buipe was one of the main centres of power of the Gonja state between the 15^th^ and the 18^th^–19^th^ centuries CE. Moreover, it was an important commercial hub on the sub-Saharan trade routes between the Niger and the tropical forest [50]. Surveys and archaeological excavations carried out between 2014 and 2022 revealed a very extensive archaeological site consisting of settlement mounds, some of them with impressive dimensions and containing imposing architectural remains [50–52]. The main occupational sequence of the site took place between the 15^th^ and the 18^th^ century CE. The 19^th^ century CE witnessed a first shrinkage in size and a shift of the settlement towards the north, where the colonial-period town flourished. By the mid-20^th^ century CE, most of the town was abandoned in favour of a new site a dozen km to the east, and only a small village remained in Old Buipe. Archaeological finds include artifacts of local and regional production (ceramic, clay smoking pipes, iron objects, etc.), as well as importation items like European clay smoking pipes and ceramics, cowries, and glass beads; with the notable exception of the glass beads, most of the importation items originates from 19^th^ and 20^th^ century CE contexts. A total of 18 glass beads found between 2015 and 2017 and coming from the earlier occupational phase were selected for techno-stylistic and chemical analysis [53]. Most of the beads are composed by multiple layers of glass for a total amount of 45 different glass samples analysed (Table 1).

**Table 1:** Summary of the glass beads collection. The summary includes the number of beads found in each site, the number of beads from dated contexts studied from a techno-stylistic point of view, and the glass samples chemically analysed.

Site	Country	Date	Glass beads found	Glass beads studied	Samples chemically analysed
**Old Buipe**	Ghana	15^th^–18^th^ c.	18	18	45
**Alinguel**	Senegal	17^th^–19^th^ c.	208	207	184
**Toumbounto**	Senegal	17^th^–19^th^ c.	690	687	561
**Farabana**	Mali	18^th^ c.	4	2	4
**Tyi-kun**	Mali	18^th^–20^th^ c.	8	2	4

#### Alinguel, Senegal

Alinguel is a large settlement site located in the Falémé River valley in Eastern Senegal (Fig 1). This site is mentioned in the 19^th^ century travelogues of various travellers and trade agents as a hinge between the regions where the Boundou and Bambouk kingdoms were established [54]. The site was excavated in 2012 and 2013, leading to the identification of three settlement areas occupied during several centuries. Radiocarbon dating allowed to determine three occupational phases, the earlier one between the 1^st^ and the 10^th^ century CE, the middle one between the 11^th^ and the 13^th^ century CE, and the latter one between the 17^th^ and the 19^th^ century CE [54]. The extensive excavation of the site delivered a considerable number of potsherds, as well as metallic objects, lithic grinding tools, botanical macro-remains, and 227 beads made of glass (N = 208), ceramic (N = 10), stone (N = 4), copper (N = 2), iron (N = 1), bone (N = 1), and indeterminate material (N = 1). Glass beads were recovered from various chronological levels of the central area of the site: 7 were found in the layers dating from the middle phase, 178 from the recent occupational levels, whereas 23 come from the surface [53]. The beads from the 11^th^–13^th^ century context resulted to be displaced from the more recent layers and were therefore included in the 17^th^–19^th^ century assemblage [53]. A total of 207 beads were included in the techno-stylistic classification, one of the beads being too degraded to be identified. Moreover, 184 glass samples composing 129 beads were selected for chemical characterization; only the beads coming from the surface and the beads presenting an advanced chemical degradation were excluded from the analysis (Table 1).

#### Toumbounto, Senegal

Toumbounto is a settlement site located on the left bank of the Falémé River in Eastern Senegal (Fig 1). As for Alinguel, this site is mentioned in several written accounts of travellers, describing it as a remarkable village linked to the Boundou kingdom in the 19^th^ century CE [54]. The site, excavated between 2012 and 2015, consists of 76 structures of various functions and one grave. Eight of these structures, namely three storage structures, two habitations, one structure linked to metallurgical activities, one grave, and one structure of unknown function, were extensively excavated [54]. Oral accounts and radiocarbon dating of several charcoal fragments and one calcinated seed led to the identification of one main occupational phase between the 17^th^ and the 19^th^ century CE linked to the architectural structures, as well as scant traces of a much older occupation around the 1^st^ century CE [54]. The archaeological finds of various nature coming from the main occupational phase include 704 beads, 690 of which made of glass, 8 made of stone, 5 made of undetermined material, and 1 made of metal [53]. A total of 687 glass beads were studied and included in the techno-stylistic classification, 426 of which were selected to be chemically analysed. These beads are composed from one to four layers of glass, for a total amount of 561 glass samples analysed (Table 1).

#### Farabana, Mali

The archaeological site of Farabana is located on the Malian side of the Falémé River Valley, 10 km east of the border between Senegal and Mali (Fig 1). The written accounts of the *Compagnie du Sénégal*, the French chartered company administering part of Senegal, mentioned the construction in 1724 of a fort near the village where the Bambouk king had his residence, precisely Farabana [55]. The excavation of the site in 2014 and 2016 led to the discovery of the ruins of this fort, which was used between 1724 and 1758 according to the texts and confirmed by the radiocarbon dating of six charcoal fragments [56: 151]. The archaeological finds include ceramics and smoking pipes of local production, cast-iron cannons, as well as surprisingly sporadic importation items, such as tableware, glass bottle fragments, and clay smoking pipes [56, 57]. Only four glass beads were recovered during excavation, one of which was found in surface and therefore excluded from the analysis. A total of 4 glass samples were chemically characterised (Table 1).

#### Tyi-kun, Mali

Tyi is a large settlement site composed by several districts located on the top of the Bandiagara Escarpment in Mali (Fig 1). Three structures from two of the districts were excavated between 2008 and 2010, specifically a habitation in Tyi-jo and two open-air pottery firing structures in Tyi-jo and Tyi-kun. The study of the archaeological finds and the oral surveys among the people living in the present village of Tyi in the plain led to the conclusion that Tyi-jo was occupied between the end of 16^th^ and the end of 17^th^ century CE, whereas Tyi-kun between the 18^th^ and mid-20^th^ century CE [58]. A total of 13 beads were recovered during the surface survey and the excavation of Tyi-kun, 8 of which made of glass. Of these, only two were found in stratigraphic position and included in the techno-stylistic and chemical study [53]. A total of 4 glass samples were chemically analysed (Table 1).

### Methods of analysis

#### Techno-stylistic characterisation

A total amount of 916 glass beads were studied macroscopically and microscopically to create a systematic classification based on morphological and technological aspects. The microscopic analysis of the beads was performed with a Leica M420 apozoom microscope equipped with a Leica DFC420 camera at the Department of Earth Sciences of the University of Geneva, as well as with an Olympus SZX10 stereomicroscope equipped with an Olympus SC50 camera at the APA Laboratory of the University of Geneva.

The parameters considered are the following:

- Size: maximum length and diameter of the bead measured with an electronic Voger Germany sliding calliper with 0.01 mm resolution.
- Shape: shape of the cross and longitudinal sections of the bead based on Horace Beck classification system [59].
- Structure: number of glass layers composing the bead.
- Colour: Munsell colour code of each glass composing the bead [60].
- Diaphaneity: level of transparency of each glass composing the bead (OP = opaque, TR = translucid, TR = transparent).
- Manufacturing technique: how the bead was manufactured (W = winding, D = drawing, M = moulding).
- Secondary modification: any intentional modification of the bead after manufacture.

#### Chemical characterisation

The 798 glass samples composing 578 monochrome and polychrome beads selected for characterisation were analysed by Laser Ablation–Inductively Coupled Plasma–Mass Spectrometry (LA–ICP–MS) at the *Institut de Recherche sur les Archéomatériaux* (CEB-IRAMAT) of Orleans in France. A Thermo Fisher Scientific ELEMENT XR mass spectrometer was used for the analysis and the glass was sampled with a Resonetics M50E excimer laser working at 193 nm and operating at 5 mJ energy and 10 Hz pulse frequency. A 20 s pre-ablation was applied to reach the unaltered bulk glass, followed by 30 s of signal acquisition, corresponding to 10 mass scans. Setting the beam diameter between 50 and 100 μm led to the creation of craters 150–250 μm deep, invisible to the naked eye. The argon/helium flow carrying the sampled aerosol to the injector inlet of the plasma torch works at a rate of 1 l/min for Ar and 0.65 l/min for He. The signal in count-per-second was measured in low-resolution mode for 58 isotopes, from lithium to uranium. Standard Reference Materials from the National Institute of Standards and Technology (NIST SRM) 610 and the Corning reference glasses B, C, and D were used for external calibration. Reference materials Corning A and NIST SRM612 were analysed with the beads as unknown samples. The in-house archaeological sample APL1 was used for chlorine quantification and ^28^Si was used as an internal standard. The protocol detailed in [61] and [62] was followed to calculate the concentration of each element, with detection limits from 0.01% to 0.1% for major elements, and from 20 to 500 ppb for minor and trace elements.

## Results

### Techno-stylistic classification

**Fig 2:**
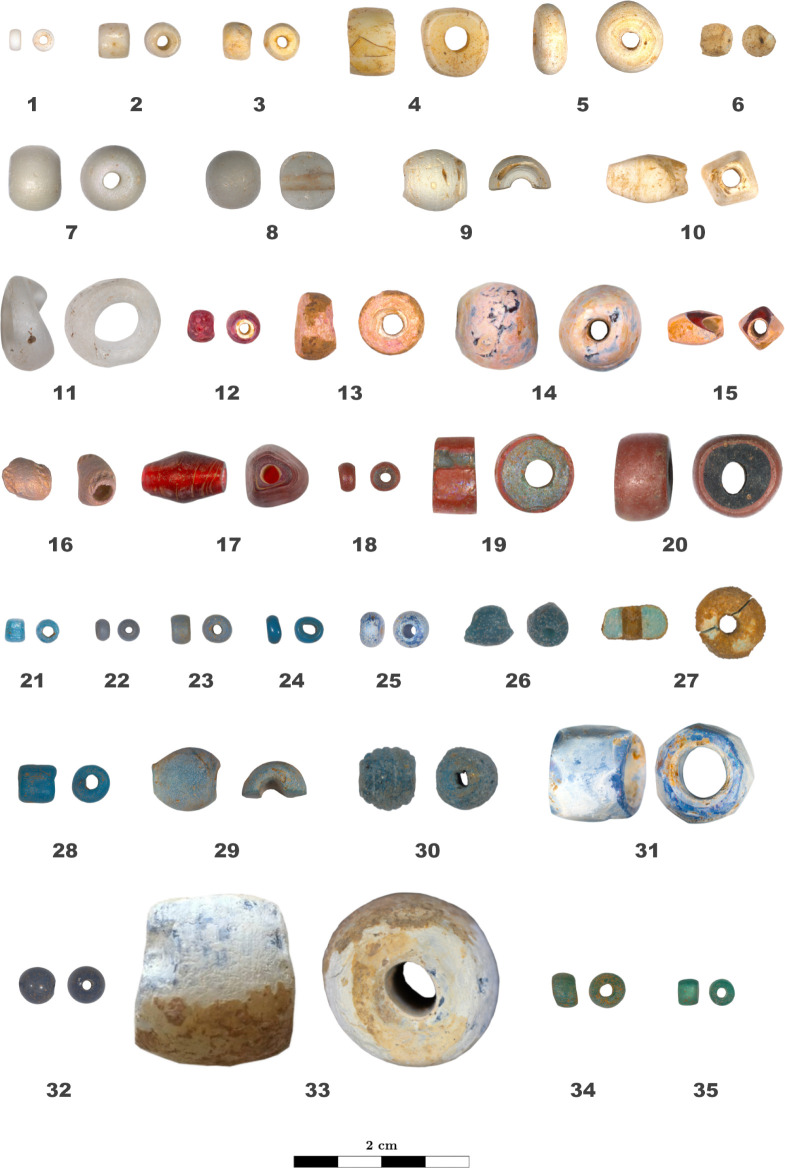
Techno-stylistic classification of the beads: Part I. Longitudinal and transversal view of bead types 1 to 35.

**Fig 3:**
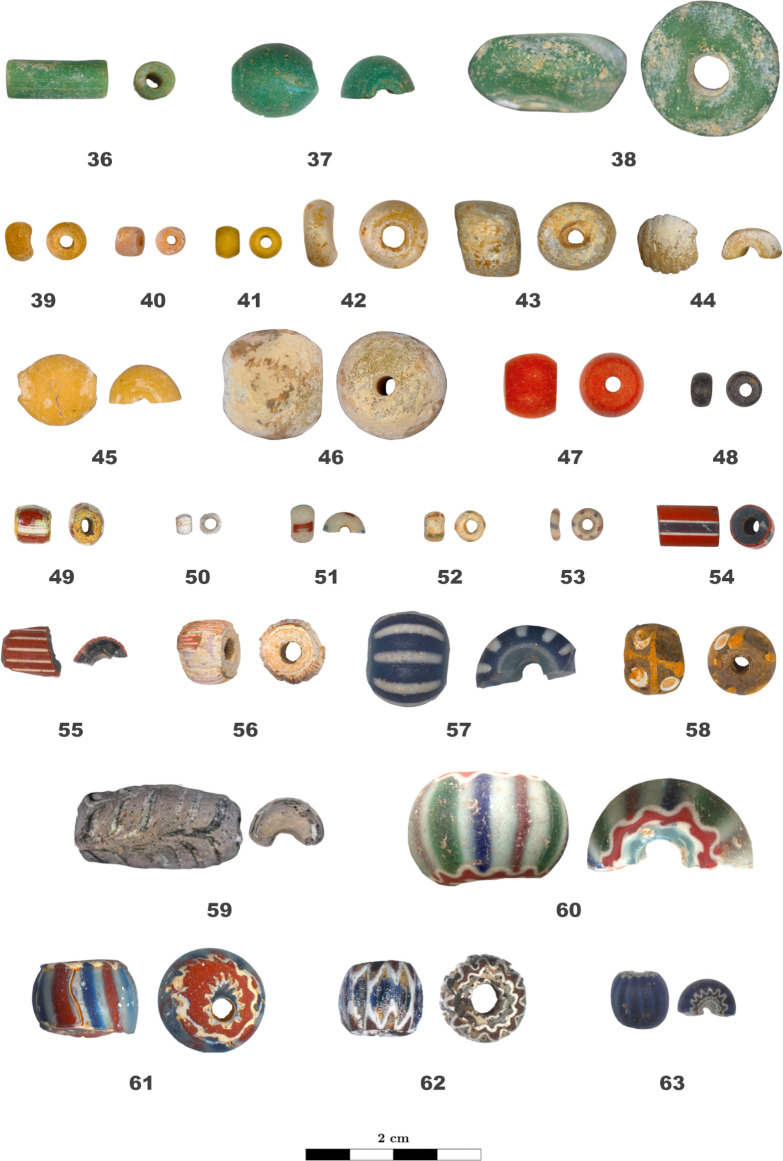
Techno-stylistic classification of the beads: Part II. Longitudinal and transversal view of bead types 36 to 63.

The macroscopic and microscopic analysis of 916 glass beads led to their classification in 63 different techno-stylistic types based on their morphology, optical properties, and manufacturing techniques (Figs 2 and 3). The beads were separated into 15 categories, namely monochrome beads of different colour (white, colourless, pink, red, green, blue, orange, yellow, and black), *Cornaline d’Aleppo*, Green Hearts, striped beads, eye beads, feather beads, and chevron beads. S1 Table lists the beads types’ morphological, optical, and manufacturing characteristics previously described.

### Chemical classification

The full chemical composition of the 798 glass samples composing the selected 578 monochrome and polychrome beads included in the techno-stylistic classification, as well as three stylistically unidentifiable fragments of beads, can be found in S2 Table.

**Fig 4:**
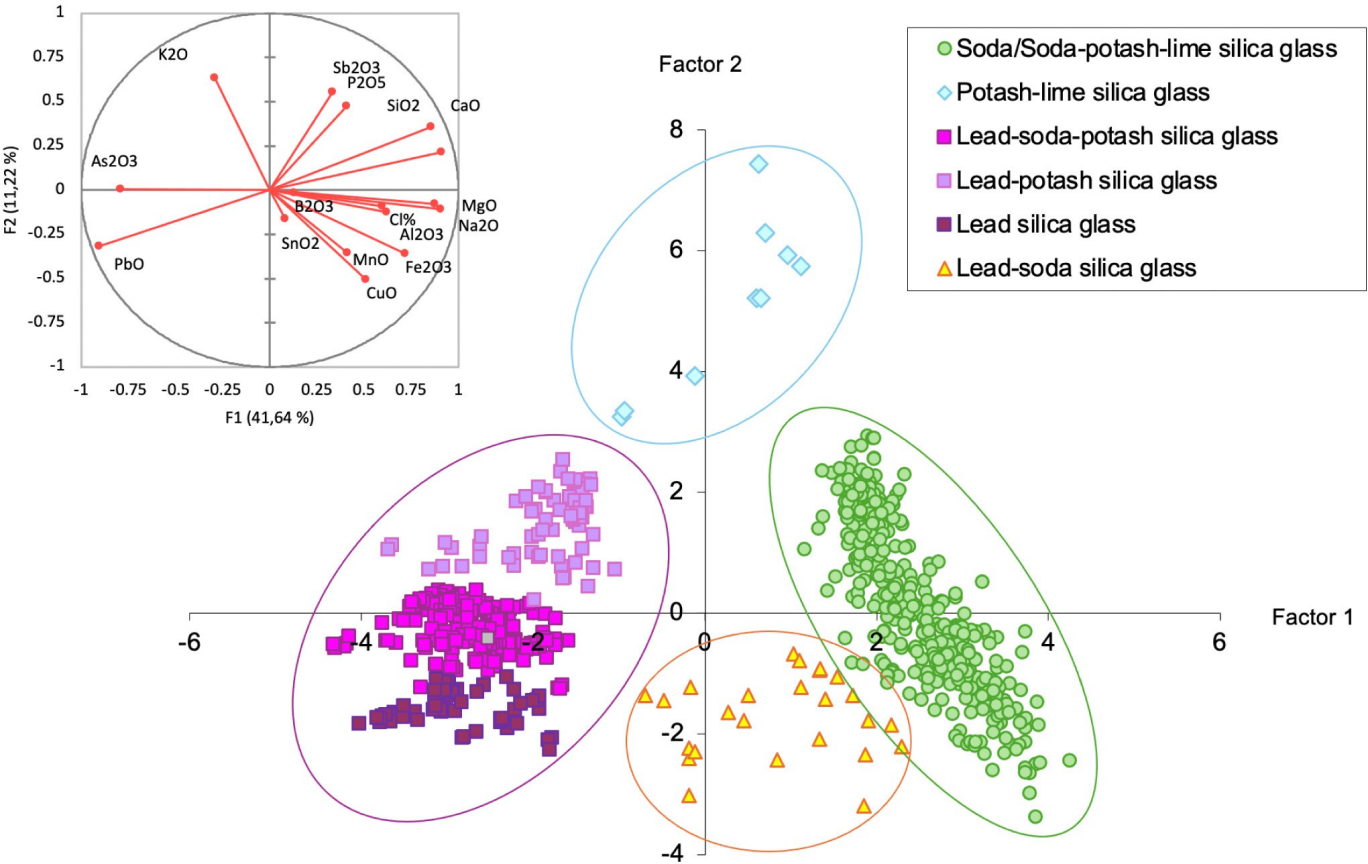
Chemical grouping. Clustering of the glass samples after PCA.

Principal Component Analysis (PCA) was applied to the collection taking into account the oxides linked to the silica source (SiO_2_, Al_2_O_3_, CaO, Fe_2_O_3_), to the flux (K_2_O, MgO, CaO, P_2_O_5_, Cl, B_2_O_3_, PbO), and to the colouring agents (PbO, Fe_2_O_3_, MnO, CuO, As_2_O_3_, SnO_2_, Sb_2_O_3_) in order to identify the main chemical groups. Keeping in mind that some of the oxides can be associated to multiple ingredients, it was possible to differentiate between four main compositional groups, *i*.*e*. soda/soda-potash-lime silica glass (S/SPL), potash-lime silica glass (PL), lead and lead-alkali silica glass (divided into three clusters depending on the alkali content: lead silica–LS; lead-soda-potash–LSP; lead-potash–LP), and lead-soda silica glass of intermediate composition (LSS) (Fig 4). The main oxide average composition of the main chemical groups can be found in S3 Table.

These groups can be further divided into 33 sub-groups considering the concentration of glass chromophores and opacifiers. It needs to be pointed out that LA-ICP-MS is an elemental analytical technique: the identification of possible chromophores and opacifiers was therefore based on bibliographic information in correlation with the elemental composition of the samples.

Table 2 lists the sub-groups of each main chemical groups indicated by the colour of the glass (blk: black, b: blue and turquoise, cls: colourless, g: green, o: orange; p: pink, r: red, w: white, y: yellow) and the chemical element characterising the colourant. The number of analysed glass samples for each sub-group, as well as the correlation between these groups and the techno-stylistic types of the corresponding beads are also indicated (cf. Figs 2 and 3, and S1 Table).

**Table 2:** Chemical classification of the analysed glass samples. Chemical grouping of the samples based on the chemical composition of the base glass and type of chromophores/opacifiers, and correlation with the techno-stylistic classification. **N:** number of samples for each group. **n/a:** stylistically unidentifiable glass beads’ fragments.

Main chemical group	Chemical sub-group	Colour	Chromophore / opacifier*	N	Techno-stylistic type(s)
Soda/Soda-potash-lime silica glass	**S/SPL blk (Mn)**	Black	Manganese oxide	13	48
	**S/SPL b1 (Co)**	Blue	Cobalt oxide	13	21, 23, 53, 54, 55, 57, 60, 61, 62, 63
	**S/SPL b2 (Cu)**	Turquoise	Copper oxide	30	21, 26, 28, n/a
	**S/SPL cls**	Colourless		15	4, 19, 20, 22, 53, 57, 60, 61, 63
	**S/SPL g (Cu)**	Green	Copper oxide	81	18, 19, 20, 49, 51
	**S/SPL r1 (Cu)**	Red	Metallic copper	87	18, 19, 20, 49, 51, 53, 54, 55, 60, 61, 62, 63
	**S/SPL r2 (Se+Cd)**	Red	Cadmium sulphoselenide	1	47
	**S/SPL w1 (Sn)**	White	Tin oxide	4	8, 51, 61
	**S/SPL w2 (SbCa)**	White	Calcium antimonate	140	3, 4, 6, 7, 18, 49
	**S/SPL w3 (**Mineral phases**)**	White	Unmelted mineral phases (?)	1	2
	**S/SPL y (Cr)**	Yellow	Chromium oxide	1	41
Potash-lime silica glass	**PL b (Co+P+Ca)**	Blue	Cobalt oxide and calcium phosphate	7	31, 33, n/a
	**PL cls**	Colourless	-	2	11
Lead-soda-potash silica glass	**LSP b (Co)**	Blue	Cobalt oxide	2	50, 59
	**LSP g (Cu)**	Green	Copper oxide	4	35
	**LSP w (AsPb)**	White	Arsenic oxide or lead arsenates	248	1, 5, 9, 10, 12, 14, 21, 50, 56, 58, 59
Lead-potash silica glass	**LP b1 (Co)**	Blue	Cobalt oxide	1	25
	**LP b2 (Cu)**	Turquoise	Copper oxide	2	29, 30
	**LP g (Cu)**	Green	Copper oxide	1	34
	**LP p (Au)**	Pink	Colloidal gold and lead arsenates	55	12, 13, 14, 15, 16, 17, 56, 58, 59
	**LP w (AsPb)**	White	Arsenic oxide or lead arsenates	7	1, 12, 13, 14
	**LP y (SnSbPb)**	Yellow	Lead antimonates and stannate	1	58
Lead silica glass	**LS b (Cu)**	Turquoise	Copper oxide	1	21
	**LS g (Cu)**	Green	Copper oxide	14	34, 36, 37, 38
	**LS o (Pb+ Impurities)**	Orange	Lead oxide and impurities (?)	42	39, 40, 42, 43, 44, 45, 46, 52
Lead-soda silica glass	**LSS blk (Mn)**	Black	Manganese oxide	2	58
	**LSS b1 (Co)**	Blue	Cobalt oxide	2	22, 32
	**LSS b2 (Co+Cu)**	Blue	Cobalt and copper oxides	3	24
	**LSS g1 (Cu)**	Green	Copper oxide	3	37, 52
	**LSS g2 (Mn+Cr+Cu)**	Green	Manganese and copper oxide, traces of chromium oxide	1	19
	**LSS g3 (Cr+Cu)**	Green	Chromium and copper oxides	1	34
	**LSS r (Cu)**	Red	Metallic copper	3	19, 52, n/a
	**LSS w (Sn)**	White	Tin oxide	10	8, 53, 54, 55, 57, 60, 62, 63, n/a

* The most likely element or oxide is proposed as the chromophore or opacifier based on the results of the elemental analysis without taking into account its oxidation state, which cannot be determined by LA-ICP-MS.

The average composition of each chemical sub-group is reported in S4 Table.

### Correlation between glass composition and bead typology

#### Monochrome beads

Monochrome beads are the most commons beads in the collection with 688 samples grouped into 39 techno-stylistic bead types, namely types 1 to 10 (white), 11 (colourless), 21 to 33 (blue), 34 to 38 (green), 39 to 46 (orange and yellow), 47 (red), and 48 (black). From a chemical point of view, there is a very good correlation between the typological classification of the beads and the chemical grouping of the glass composing them; beads within the same type have in fact the same base composition, except for type 21 and 34 including beads with 3 different glass compositions each and type 37 with 2 compositions. Several bead types, on the other hand, might fall into the same chemical group.

The majority of the monochrome white beads are made of potash and mixed-alkali lead glass opacified either by arsenic oxide or by a compound based on lead arsenates; they were found in Alinguel, Toumbounto, and Farabana (N = 257; groups LSP w and LP w; types 1, 5, 9, 10). The second most common monochrome white beads are composed by soda-lime silica glass opacified by calcium antimonate forming white crystals embedded in the glass matrix, and they were found in Alinguel, Old Buipe, and Toumbounto (N = 140; group S/SPL w2; types 3, 4, 6, 7). Three glass beads from Old Buipe are made of soda-lime lead-silica glass and probably opacified by tin oxide by precipitation of small cassiterite particles during cooling (N = 3; S/SPL w1; type 8). Finally, bead TO-52-5w from Toumbounto has an alumina-silica glass matrix (10.6 wt% of Al_2_O_3_), is fluxed with soda and potash and contains low amounts of P, As, Sn and Sb (N = 1, S/SPL w3, type 2).

The two colourless translucent glass beads from Alinguel analysed (type 11) are composed by potash lime silica glass containing 17–18% of K_2_O (group PL cls).

Blue and turquoise monochrome beads are the most variable both from a techno-stylistic and a chemical point of view (48 beads, 13 types, 8 groups). Most of these beads were found in Old Buipe and Toumbounto and are composed by soda-lime silica glass containing between 10 and 18 wt% of soda, between 1.2 and 6.4 wt% of potash, and between 1.4 and 2.2 wt% of copper oxide (N = 30, group S/SPL b2, types 21, 26, 28). Three other beads from type 21, together with the only bead from type 23, are also composed by soda-lime silica glass but they contain a lower amount of soda (10 wt%), higher amount of antimony (4–9 wt%), and they are coloured with cobalt. These beads were all found in Toumbounto (N = 4, group S/SPL b1, types 21, 23). Finally, one last bead from type 21 found in Toumbounto is composed by low alkali lead glass (60 wt% of PbO) coloured by copper oxide (group LS b). The high variation in composition of type 21 beads might be due to the alteration of the glass, which affected the identification of their typology. The beads presenting a surface alteration layer were in fact only partially cleaned to reveal the unaffected glass underneath. Six multifaceted monochrome blue beads are made of potash-lime silica glass coloured by cobalt and opacified by calcium phosphate, and they were found in Alinguel, Toumbounto, and Tyi-kun (group PL b, types 31 and 33). Finally, eight monochrome blue beads are composed by alkali lead silica glass and are either fluxed with soda and coloured by cobalt and containing tin (N = 2, group LSS b1, types 22, 32, from Old Buipe) or cobalt and copper containing arsenic (N = 3, group LSS b2, type 24, from Toumbounto), or fluxed with potash and coloured by cobalt (N = 1, group LP b1, type 25, from Toumbounto) or by copper oxide (N = 2, group LP b2, types 29 and 30, from Alinguel and Toumbounto). The three beads from type 24 show a very high alumina content.

Monochrome green beads are mainly made by low alkali lead glass despite the variability in shape and size (N = 14, group LS g, types 34, 36, 37, 38). Four beads from type 35 are made of soda-potash silica glass coloured by copper oxide (group LSP g). Besides, fewer beads are made by lead-soda silica glass, two of which coloured by copper oxide (N = 2, group LSS g1, types 37) and one coloured by chromium and copper oxides (N = 1, group LSS g3, type 34). Finally, one other bead from type 34 is a lead-potash silica glass coloured by copper oxide (N = 1, group LP g, type 34). All monochrome green beads were found in Alinguel and Toumbounto.

Similar to the green beads, all orange monochrome beads have the same base glass composition despite the variability in typology, and they were found in Alinguel and Toumbounto. They are composed by low alkali lead glass, which has a typical natural yellow-orange hue, in this case probably reinforced by the presence of low amounts of antimony and possibly lead antimonates, as well as other impurities (N = 42, group LS o, types 39, 40, 42, 43, 44, 45, 46). Besides, one yellow bead from type 41 is composed by mixed-alkali alumina-silica glass coloured by chromium (group S/SPL y).

The only monochrome red bead of the collection was found in Toumbounto and it is composed by alumina-silica glass fluxed with soda, coloured by cadmium sulfoselenide, and containing 5 wt% of boron (group S/SPL r2, type 47). It is interesting to notice that a fragment of glass with the same composition was found in Dourou-Boro, an archaeological site not far from Tyi-kun in Mali, in a context dating from the 7^th^–9^th^ century CE [4]; given that this chromophore was used only after the 19^th^ century CE [63, 64], this fragment was interpreted as the result of looting of the funerary site.

Finally, all the 13 monochrome black beads found have the same typology and are composed by soda and soda/potash lime silica glass and are coloured by concentration of manganese oxide between 3.5 and 6.6 wt% (group S/SPL blk, type 48). It is interesting to notice two slightly different compositions in relation to the site of discovery, possibly suggesting two different bead suppliers. As a matter of fact, the 7 beads from Alinguel plus one from Toumbounto contain higher amount of soda (12–16 wt%) and antimony (0.3–0.9 wt%), and lower amount of potash (0.8–1.9 wt%), whereas the beads found in Toumbounto except one are composed by mixed-alkali silica glass (soda around 8 wt% and potash around 5 wt%) with trace amount of antimony.

#### Cornaline d’Aleppo

The so-called *Cornaline d’Aleppo* are the most common beads found in Alinguel and Toumbounto after the white monochrome beads (N = 122). These beads generally comprise a translucent pink ruby glass layer on the exterior and an opaque white one on the interior (types 12, 13, and part of 14). However, the white layer might not be present in reason of the complete degradation of the glass or for the technical choice of the glassmaker (types 15 to 17, and part of 14).

**Fig 5:**
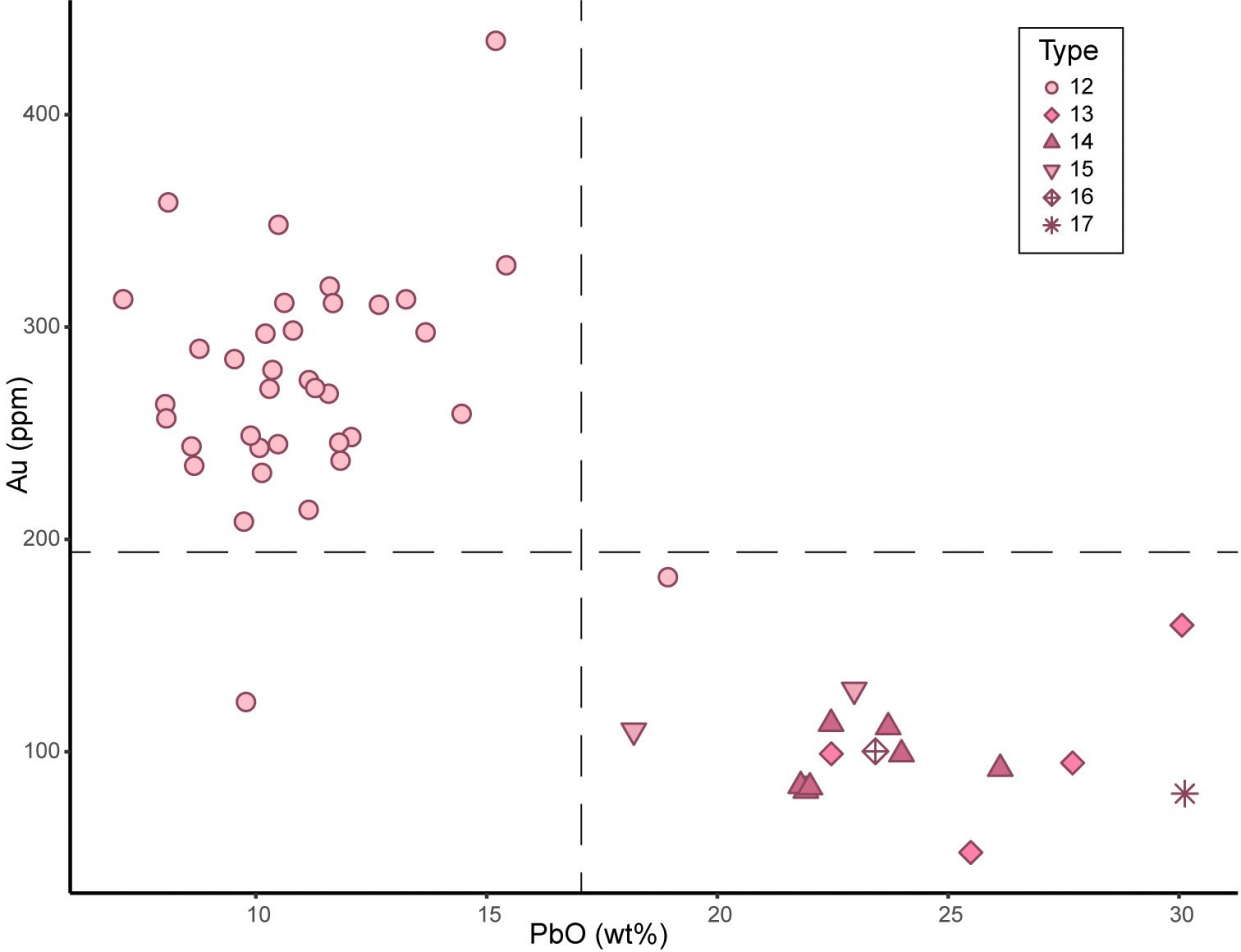
Red glass of Cornaline d’Aleppo. Correlation between lead oxide and gold concentrations in the pink ruby glass composing the Cornaline d’Aleppo found in Alinguel and Toumbounto. The composition is consistent with the typology of the beads.

The ruby glass composing all *Cornaline d’Aleppo* analysed is lead-potash silica glass containing 8 to 15 wt% of potash and 3 to 7 wt% of soda. The colouring agent of this glass is colloidal gold in concentrations between 53 and 435 ppm (N = 50, group LP p, types 12 to 17). The technology for creating ruby red glass through metallic gold is ancient and involves the precipitation of Au(0) nanoparticles though addition of tin salts, iron oxides or, more recently, arsenic salts as reducing agents [49, 65–67]. The high amount of arsenic present in this glass suggests the use of an arsenic compound together with gold to obtain the ruby colour, as described in Venetian recipes dating from the end of the 17^th^ century [68]. It is possible to see a pattern in the amount of gold and lead oxide in relation with the typology of the beads. Type 12 shows in fact (with one exception) lower amount of PbO (11±2 wt%) and higher amount of Au (275±52 ppm), whereas types 13 to 17 show higher concentration of PbO (24±3 wt%) and lower concentration of Au (105±32 ppm) (Fig 5).

The potash concentration is also different in the two groups whereas the trace elements content is the same, meaning that the same raw materials were used for production, but different alkali and lead oxides were introduced, probably to optimise the malleability in relation to the fabrication technique.

Beads from types 12, 13, and few from type 14, show a layer of opaque white glass having the same composition as the monochrome white beads from types 1, 5, 9, and 10. It is a lead-potash or lead soda potash silica glass opacified either by arsenic oxide or by a compound based on lead arsenates (N = 46, groups LSP w and LP w).

#### Green Hearts

**Fig 6:**
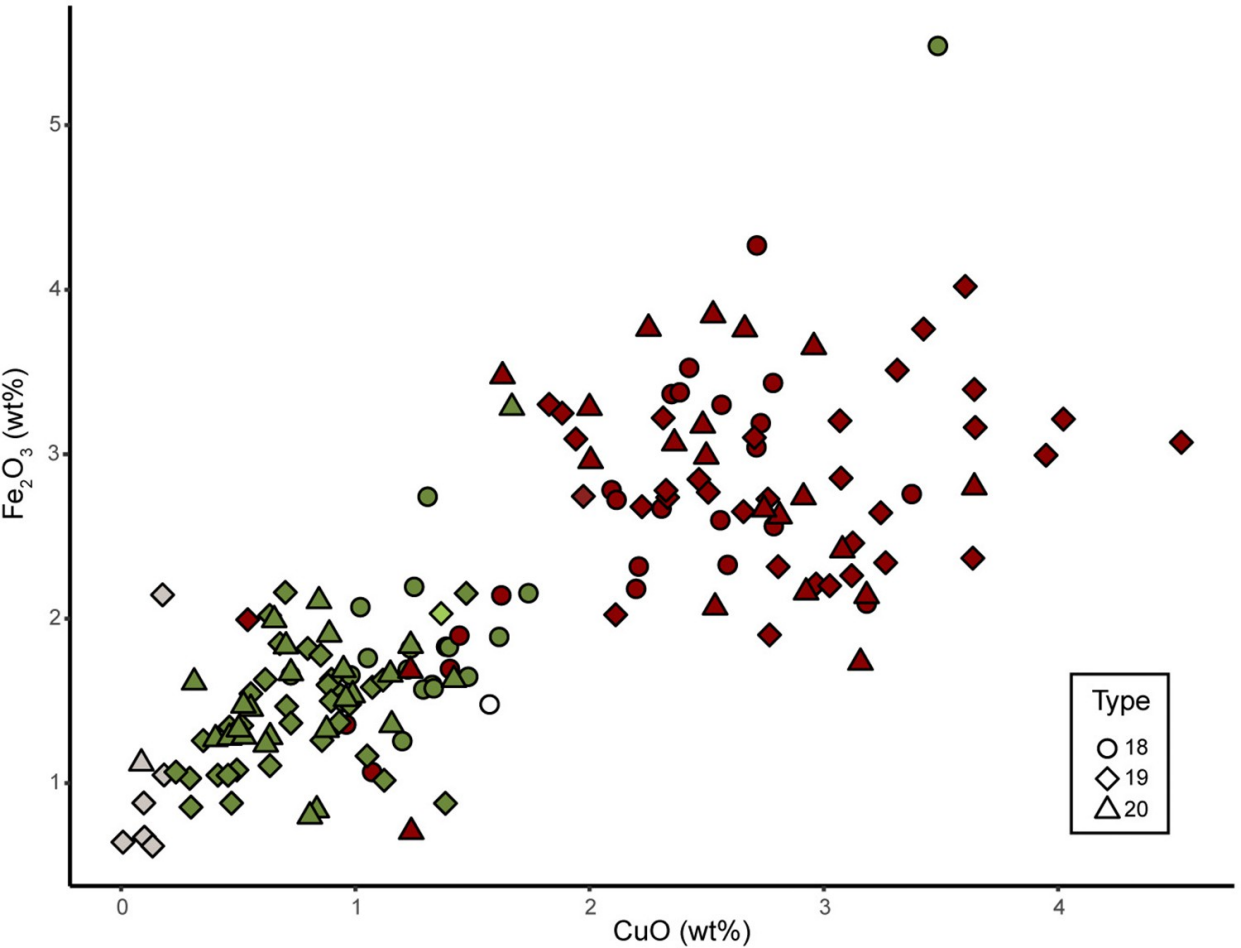
Green Hearts composition. Copper and iron oxides concentrations of the green, red, and colourless glass composing the Green Hearts found in Toumbounto, Alinguel, and Farabana. The colour of the points corresponds to the colour of the glass.

The so-called Green Hearts are generally composed by two layers of glass, one opaque carmine red on the exterior and one transparent to translucent, colourless to green one on the interior. Few beads also show a transparent colourless glass layer on the external surface. The 88 Green Hearts found in Alinguel, Toumbounto, and Farabana can be divided into three types, from 18 to 20, based on their size and finish (S1 Table). A total of 163 glass samples were chemically analysed, 79 of which are green, 82 are red, and 7 are colourless. All glass samples, except the ones composing one bead from type 19, show the same base composition, *i*.*e*., soda-lime silica glass with around 13 wt% of soda, 3 wt% of potash, and 10 wt% of lime. The only factor changing is the amount of copper and iron oxides giving colour to the glass (Fig 6).

The colourless layer of glass on the surface of the beads has negligible amount of copper and iron oxides (N = 7, group S/SPL cls, types 19, 20). On the other hand, the 82 red glass samples from group S/SPL r1 show higher amount of CuO (2.5±0.8 wt%) and Fe_2_O_3_ (2.7±0.8 wt%), whereas the 79 green glass samples from group S/SPL g contain 0.9±0.8 wt% of CuO, 1.6±0.4 wt% of Fe_2_O_3_, and varying amount of antimony oxide (between 0.1 and 3.2 wt%), in relation with the opacity of the glass. It is difficult to understand the nature of the glass chromophore without a molecular analysis of the glass. However, the different amount of copper and iron oxides could explain mechanisms of red glass colouration. As the green colour is generally given by CuO, the red colour could be either given by Cu_2_O or metallic Cu particles in suspension [49, 67, 69]. It has been proven that in red glass containing copper oxide in concentration higher than 5 wt% and high amount of lead oxide, the formation of Cu_2_O crystals is favoured [70, 71], which is not the case for the analysed samples. Besides, the high concentration of iron oxide in our samples can explain the precipitation of the metallic copper particles in the glass, as it could work as a reducing agent for the copper oxide [67, 71].

The only Green Heart with different composition is from type 19 and was found in Toumbounto; it is composed by lead-soda silica glass, the red layer probably coloured by metallic copper and having high alumina concentration (4.4 wt%, group LSS r), and the green layer showing high manganese and copper concentration, as well as traces of chromium oxide (group LSS g2).

#### Striped beads

Eleven striped beads (types 49 to 57) were found in Alinguel, Old Buipe, and Toumbounto, and they are composed by a variable number of glass layers. A total of 31 glass samples were chemically analysed.

The two beads from type 49 found in Toumbounto are visually similar to the Green Hearts from the same site but they have in addition white stripes on the surface. The green core and the red layer have the same composition as the other Green Hearts (groups S/SPL g and S/SPL r), whereas the white stripes are made of soda-lime silica glass opacified by calcium antimonate (group S/SPL w2).

Two beads from type 50 have a blue core made of lead-soda-potash silica glass coloured by cobalt (group LSP b) and white stripes made of lead-soda-potash silica glass opacified either by arsenic oxide or by a compound based on lead arsenates (group LSP w).

Beads from types 51, 53, 54, 55, and 57 were found in Old Buipe and, despite having different typologies, they show the same glass composition for each colour present. All white layers are composed by lead-soda silica glass opacified by tin oxide, as are the monochrome white beads found in the same site (group LSS w). Besides, the white glass from type 51 show lower amount of lead and falls into the group S/SPL w1. The red and the blue layers from all types are composed by soda-lime silica glass coloured respectively by metallic copper (group S/SPL r1) and by cobalt (group S/SPL b1), both opacified by tin oxide in varying amount in correlation with the lead oxide concentration. The one bead from type 53 shows two colourless layers of soda-lime silica glass, the interior one containing around 3 wt% of SnO_2_ and 7 wt% of PbO, in line with most of the glasses from Old Buipe (type S/SPL cls), and the exterior one showing on the contrary negligible quantities of these two oxides. The latter colourless glass was also found in the bead from type 57. Finally, the green stripes of the bead from type 51 are compose by soda-lime silica glass coloured by copper oxide (group S/SPL g).

The striped bead from type 52 found in Toumbounto is composed by a core of orange lead silica glass (group LS o) and red and green stripes of lead-soda silica glass coloured by copper (groups LSS r and LSS g1).

Finally, the striped bead from type 56 found in Alinguel has the same composition as the *Cornaline d’Aleppo*, with the red lead-potash glass coloured by gold (group LP p) and the white lead-soda-potash silica glass opacified either by arsenic oxide or by a compound based on lead arsenates (group LSP w). The Zr and Nd content of this glass is also comparable to the other gold ruby glass, indicating a similar origin of the raw materials used for production.

#### Eye beads

Two eye beads were found in Toumbounto (type 58), and they are composed by a black glass core and concentric yellow, pink, and white spots on the surface. The chemical analysis showed however that the yellow glass is present only in one of the beads, and it is a lead-potash silica glass coloured by lead antimonates and stannate (group LP y). The black glasses of the two beads are lead-soda silica glass coloured by manganese, showing however different amount of lime (group LSS blk). The pink and the white glasses are present in both beads, and they have the same composition as the glasses composing the *Cornaline d’Aleppo*, *i*.*e*. lead-potash silica glass coloured with gold (group LP p) and lead-soda-potash silica glass opacified either by arsenic oxide or by a compound based on lead arsenates (group LSP w).

#### Feather beads

The feather beads decoration is made by winding rods of different glass colour around the bead core and then creating a pattern by dragging the trails in different directions [72].

One feather bead (type 59) was found in Tyi-kun and it is composed by three different glasses: a pink core made of lead-potash silica glass coloured by gold (group LP p), dark blue stripes made of lead-soda-potash silica glass coloured by cobalt (group LSP b), and white lines made of lead-soda-potash silica glass opacified either by arsenic oxide or by a compound based on lead arsenates (group LSP w). The blue glass composing this bead has the same composition as the other blue bead found in Tyi-kun. The composition of the white, pink, and blue glass is comparable to the glass composing the *Cornaline d’Aleppo* and the monochrome white and blue beads from the same chemical groups, showing however in each case a higher content of Zr and suggesting therefore the use of a different kind of sand for production.

#### Chevron beads

Chevron beads were created by blowing a mass of glass into a star-shaped mould, repeating the process layer after layer with different coloured glass. After drawing a glass cane and cutting the singular pieces, the layers were revealed by mechanical abrasion of the extremities of each bead [20, 72, 73].

**Fig 7:**
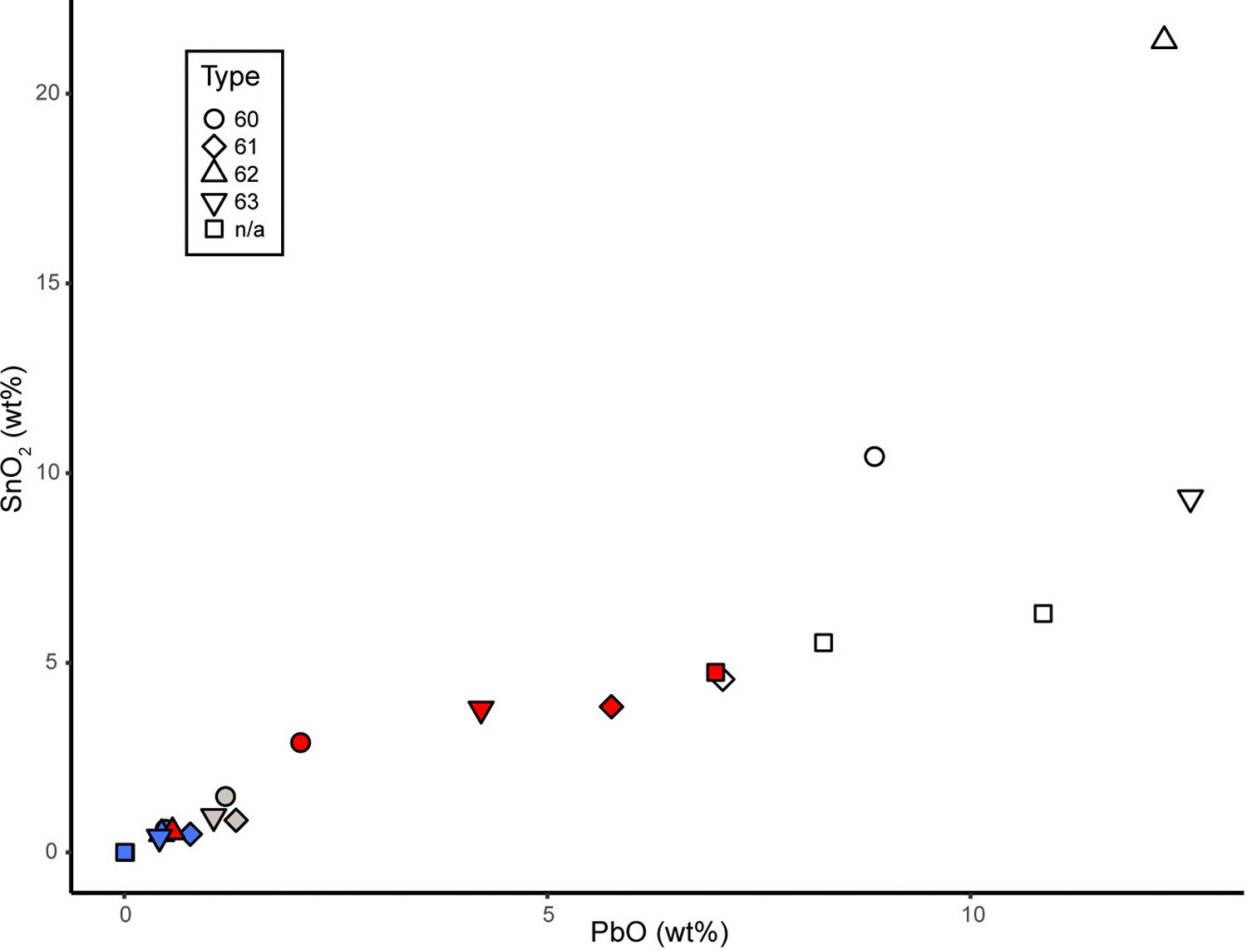
Chevron beads from Old Buipe. Correlation between tin and lead oxides in the glass layers composing the chevron beads found in Old Buipe. The colour of the points corresponds to the colour of the analysed glass.

The four chevron beads found in Old Buipe show different number of layers and colours (types 60 to 63), like the very degraded red and white chevron bead not included in the techno-stylistic classification (n/a) (Table 2). Besides, the composition of the glass of each colour is the same of the ones composing the other beads from this archaeological site. The white layers of types 60, 62, 63, and “n/a” are made of lead-soda silica glass opacified by tin oxide (N = 4, group LSS w), whereas the white glass from type 61 is a soda-lime glass opacified by tin oxide (N = 1, group S/SPL w1); the red, blue, and colourless glass samples of all chevron beads have a soda/soda-potash-lime-silica base composition and they are coloured respectively with metallic copper (N = 6, group S/SPL r1), cobalt (N = 4, group S/SPL b1), and various amount of tin oxide depending on the opacity (N = 3, group S/SPL cls). All glasses show a positive linear correlation between Sn_2_O and PbO, indicating the probable use of lead-tin calx to opacify the matrix as for most of the other beads found in Old Buipe (*see infra*) (Fig 7) [74].

## Discussion

### Hypothesis on the provenance of the beads

Many of the analysed beads coming from the excavated sites in Ghana, Senegal, and Mali with a specific typology can be associated to well-known European productions thanks to historical documents, travel accounts, and trade reports describing and classifying these items. The *Cornaline d’Aleppo*, also known as white hearts or Hudson Bay beads, for example, were produced from the beginning of the 19^th^ century in Venice and can be found, among others, in the catalogues of the Levin Collection (1851–1863), displaying the beads typologies produced specifically for the trade with West Africa [18, 19]. In these catalogues, it is also possible to find the Green Hearts, or pre-white hearts, exchanged since the 17^th^ century [19, 21], which are also displayed in the sample cards of the Venetian society *Società Veneziana per l’industria delle conterie* and of the Amsterdam’s factory J.F. Sick & Co. dated from the end of the 19^th^ and the beginning of the 20^th^ century [19]. The samples cards from these two societies exhibited in addition a great variety of monochrome beads, also known as seed beads or *rocailles*, largely present in our collection. Chevron beads, also known as *rosetta beads*, were originally produced in Venice in the 15^th^ and 16^th^ century and later imitated by other production centres in the Netherlands, Germany, and in North America. All chevron beads patterns from our collection can be found in the catalogues of chevron beads for the West African trade created by John and Ruth Picard [20, 21], as well as in the private collection of Cesare Moretti [75]. In the Picard catalogues, it is also possible to find a good match for our blue multifaceted beads known as *Russian Blues* [76], produced originally in Bohemia after the 18^th^ century CE [47, 72, 77, 78]. The eye beads and the feather bead from our collection can also be found in these catalogues (respectively on page 21 of [76] and page 3 and 32 of [20].

Many types of beads from our collection can also be matched to glass beads found in several archaeological sites in sub-Saharan West Africa [7–13]. However, few of these assemblages were chemically analysed. The typological analysis of the beads alone is in fact not enough to pinpoint the production sites of the glass and the artifacts. If Venice and Murano were the main European production sites between the 13^th^ and the 16^th^ century CE, many expert artisans started to migrate towards northern Europe to create new production centres, while Venice still remained very productive [17, 46, 47, 79, 80]. The chemical analysis of the glass composing the beads might help distinguish between different productions dated 17^th^ to 20^th^ century CE, although it still remains a complicated task, as the glass recipes and raw materials used in different glasshouses were often extremely similar, if not the same. As of today, no systematic chemical analysis of the glass beads exhibited in the commercial catalogues has been done, so we can rely for the provenance study solely on the characterisation of glass artefacts and production waste found in archaeological sites in Europe [81–86], North America [87–95], and sub-Saharan Africa [10, 96–100]. Furthermore, the approximate chemical composition of various 15^th^–20^th^ century glass types calculated from multiple Venetian recipe books can be used for comparison [48, 74, 75, 101–103].

In order to evaluate the possible provenance of the glass samples, the four main compositional groups discussed above, namely soda/soda-potash-lime silica glass, potash-lime silica glass, lead and lead-alkali silica glass, and lead-soda silica glass of intermediate composition (Fig 4, Table 2, and S3 Table), were taken into account. To simplify the provenance study, some of the glass samples of intermediate composition were included in the discussion either of the soda/soda-potash-lime silica glass or the lead and lead-alkali silica glass.

#### Soda/soda-potash-lime silica glass

The white bead from type 2, the yellow bead from type 41, and the red bead from type 47 found in Toumbounto show very high levels of alumina (10.5–13.7 wt%), suggesting that they are faïence, sintered glass or Prosser-molded glass or ceramic beads probably produced after the 19^th^ century [63]. This hypothesis is supported by the modern colourants detected, *i*.*e*. the Imperial Red (cadmium sulfoselenide), and the chromium yellow, used as glass chromophores in Venice starting from the second half of the 19^th^ century [48, 63, 92, 104]. Since no classical opacifying agents (e.g. Sb, Sn, or As compounds) were detected, the opacity of these beads is probably due to the presence of unmelted minerals in the glass matrix, although this should be confirmed by the molecular analysis of the glass.

**Fig 8:**
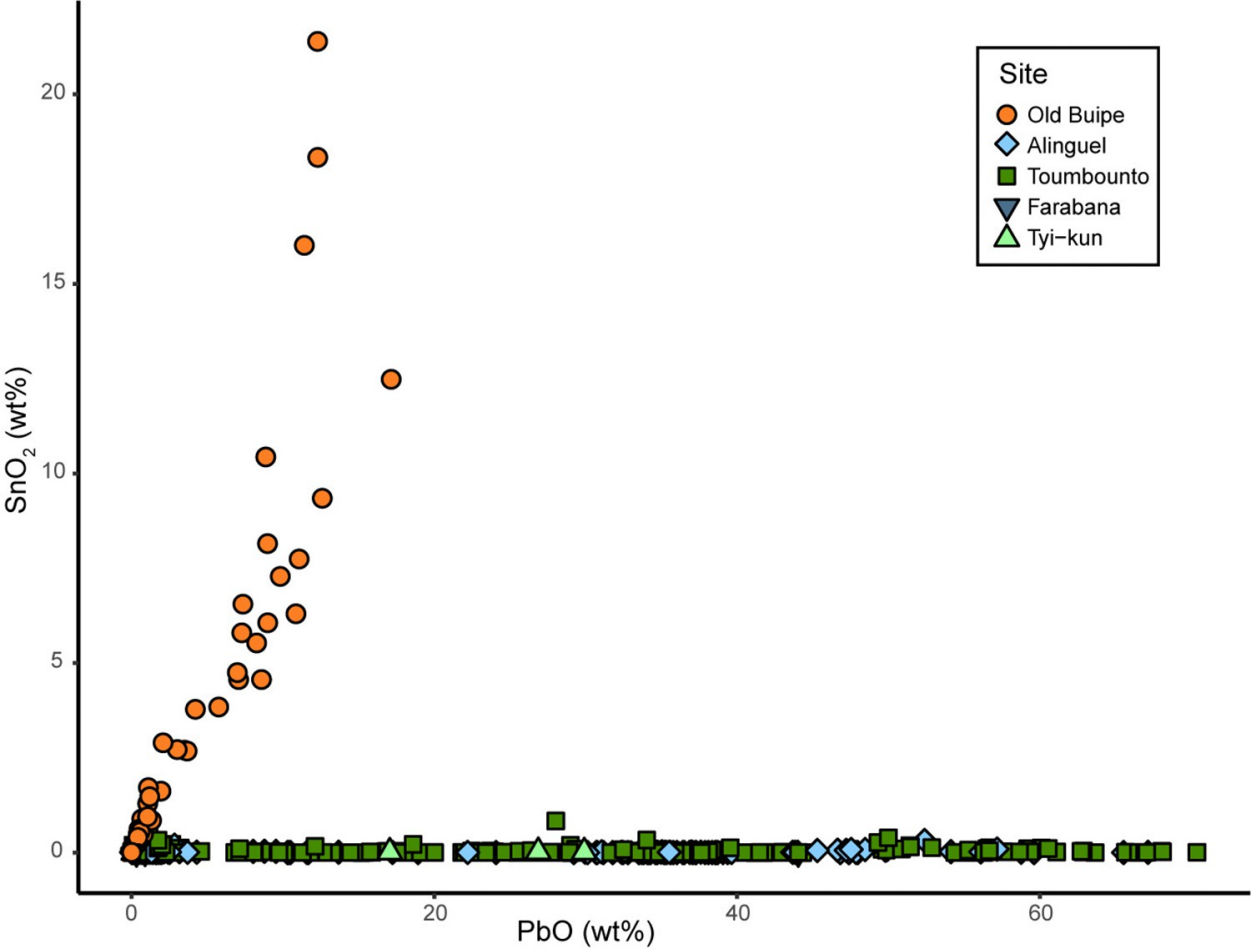
Subgrouping of soda-lime silica glass samples. Correlation between lead and tin oxides for the soda-lime silica glass samples from the different sites allowing to separate the samples into two sub-groups.

The other alkali-lime silica glass samples can be further divided in two subgroups in relation to the tin and lead oxide contents (Fig 8).

The majority of the glass samples composing Old Buipe beads shows a positive correlation between lead and tin, suggesting a coherence in the glass supply and indicating the use of the so-called *calce di stagno e piombo* (lead-tin calx) for opacification, a method used by Venetian glassmakers until the 19^th^ century [74]. Moreover, the high Sn/Pb content in the glass due to the use of tin as opacifier suggests a production predating 1650, after which the use of antimony as an opacifier became systematic [84]. This is coherent with the results of the study of North American archaeological bead assemblages of probable Dutch origin, for which the tin content is used as a chronological marker in opposition to antimony and arsenic [89, 92, 93]. The composition of Old Buipe glass is comparable to the chemical group 2 found in the North American sites of Petun and Seneca, although with lower levels of Na_2_O and CaO [93].

**Fig 9:**
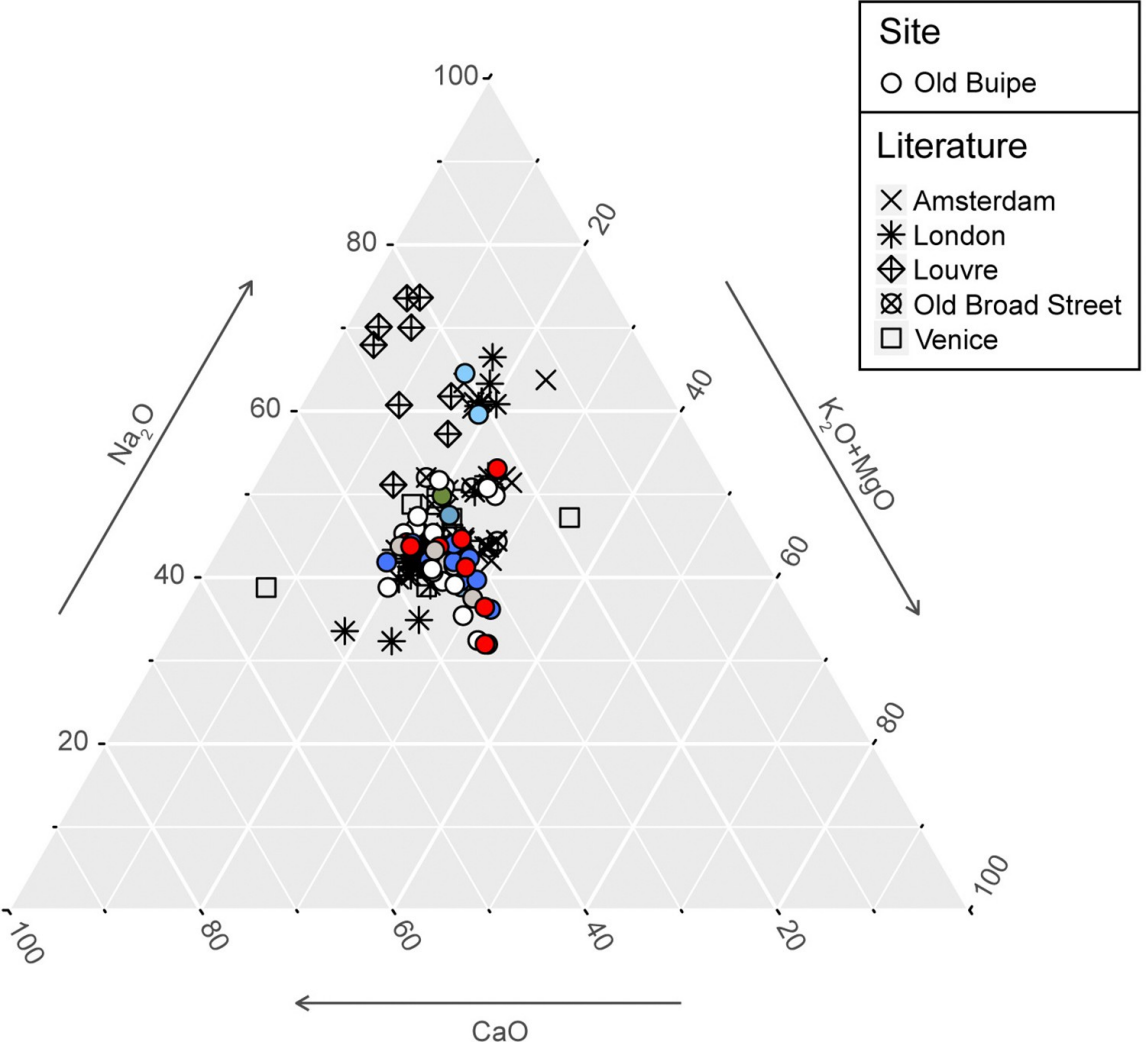
Old Buipe glass. Comparison between Old Buipe glass composition and the composition of glass from Amsterdam and London [82, 105], Old Broad Street, London [106], Louvre [103], and Venice [75].

Old Buipe glass samples have the typical composition of tin-opacified *lattimo* glass produced in Venice between the 15^th^ and 17^th^ century [75]. The comparison with the composition of glass assemblages from European archaeological sites and museum collections shows that they are comparable with the glass beads found in the late 17^th^ century Hammersmith Embankment site in London (chemical group 1) and the glasswork waste from the contemporary Kg10 production site in Amsterdam [82, 105] (Fig 9).

Moreover, compositional and typological similarities can be found between Old Buipe chevron beads and the chevron beads found in the 16^th^–19^th^ century glasswork site of Neularten, Germany, probably imported from Venice or Amsterdam [83, 84].

**Fig 10:**
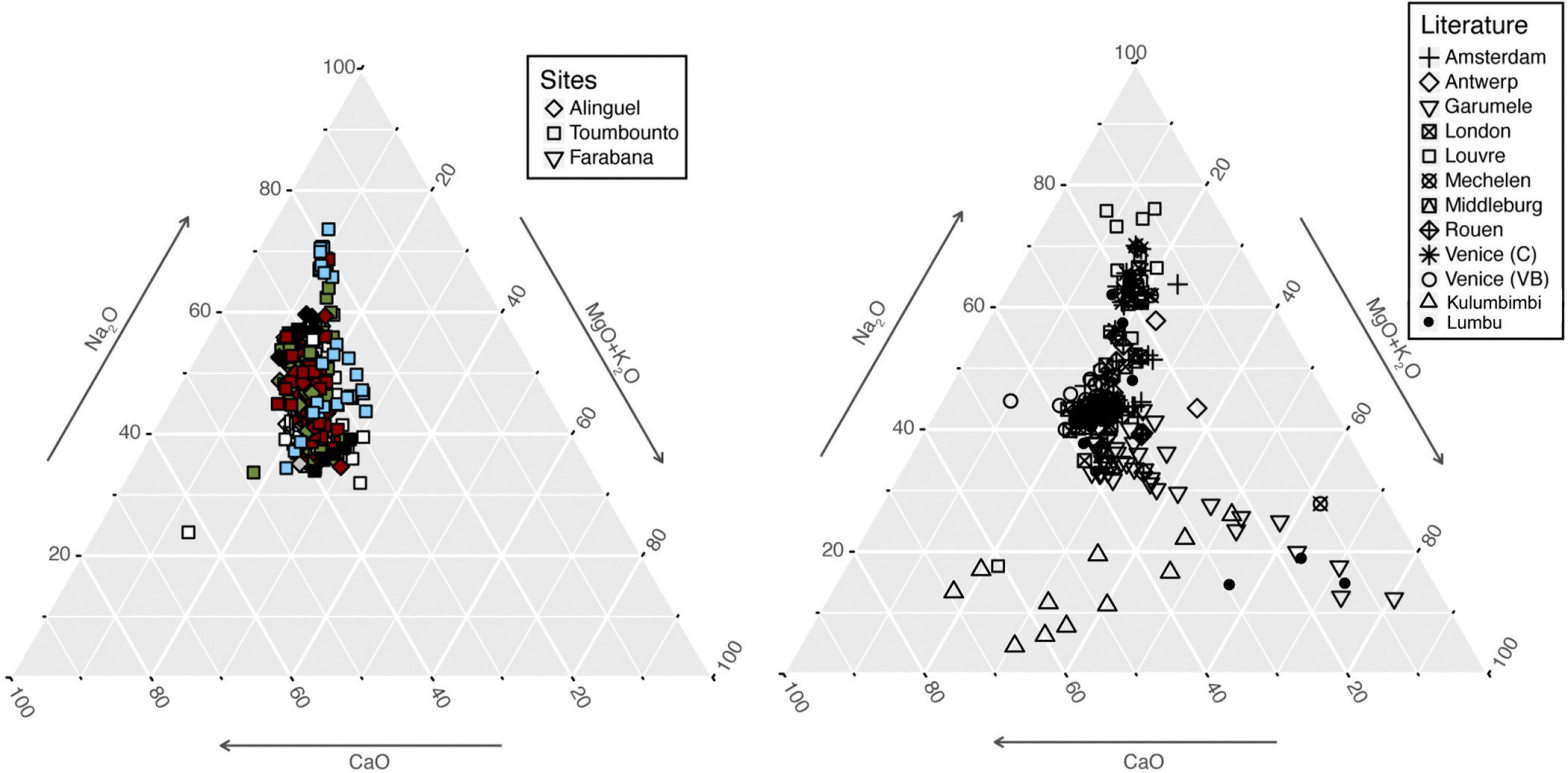
Soda and soda-potash-lime silica glass beads from Alinguel, Toumbounto, and Farabana. Comparison between mixed alkali and soda-lime glass composition and the composition of glass from Amsterdam and London [82], Antwerp, Mechelen [86], Garumele [10], Louvre [103], Middleburg [85], Rouen [81], Venice [101], and Kulumbimbi and Lumbu, Angola [98, 99]. For Venetian glass: C = Cristallo, VB = Vitrum Blanchum.

Concerning the soda and soda-potash-lime silica glass beads from Alinguel, Toumbounto, and Farabana, the composition of contemporary European and African archaeological glass and glass beads assemblages can be used to understand the possible origin of these beads (Fig 10).

The base glass of our assemblage can be compared to most archaeological European glass assemblages for which a Venetian production is suggested, as well as to the *Cristallo* and *Vitrum Blanchum* glass produced in Venice between the 15^th^ and the 17^th^ century [101]. Some of our beads, namely few turquoise beads from type 21 and the red and green glass composing some Green Hearts from type 18 and 19, have lower potash, magnesia, lime, and alumina content, much like *Cristallo* glass produced with purer sources of silica. However, unlike *Cristallo*, they contain higher amount of phosphorus, chlorine, and iron, especially the Green Hearts.

The other glass samples, more similar to the *Vitrum Blanchum* references, show higher amount of potash, much like the glass samples from 17^th^ century Mechelen and Antwerp, in Belgium [86]. Here, to explain this difference in K_2_O content, the authors suggest a production in the Low Countries instead of Venice, which is supposedly supported by the trace element content. Unfortunately, the trace element concentrations were not published, so it is not possible to compare them to the composition of our samples. However, a higher potash content is typical of Venetian glass produced between the 16^th^ and 18^th^ century adding to the glass matrix the so-called *grepola*, the deposit of the bottom of the wine barrel (potassium bitartrate). The higher potassium oxide content could be also explained by the addition of tartar (K-bearer) to the glass matrix, practice more and more common from the 17^th^ century onward [46, 107].

It is interesting to notice that techno-morphologically consistent soda-lime glass beads from Garumele, the only contemporary assemblage from a West African site to be chemically analysed, show lower concentrations of soda and lime, as well as higher concentration of potash and alumina compared to our beads, so a different production site is probable (Fig 10).

Moreover, comparing our assemblages to northern American assemblages, a parallel can be found with the soda-lime beads from 18^th^–19^th^ century Sullivand’s Island attributed to the Venetian production, although the latter show in average lower alumina content [94]. Even though beads from other North American sites [87, 89–93] show lower soda and lime content and higher potash and alumina content, like Garumele beads, the opacifier content, especially for white beads, can be used as an indicator of possible production. As already mentioned, tin, antimony, and arsenic were used as chronological markers for North American bead assemblages of possible Venetian or Dutch production, dating antimony opacified beads between the 17^th^ and the 19^th^ century [89, 92]. Considering that Amsterdam stopped to be an important glass beads production centre after the beginning of the 18^th^ century [94], we can suggest that the mixed alkali and soda-lime beads from our assemblage are of Venetian manufacture, probably dating between the late 17^th^ and the 19^th^ century.

#### Potash-lime silica glass

This group includes the glass composing the colourless monochrome beads (type 11), the blue monochrome beads from types 31 and 33, and one blue glass sample not included in the techno-stylistic analysis (TO-29-80-3b). All beads were found in Alinguel and Toumbounto, Senegal. Their high content in potash and phosphorus corresponds to the use of forest wood ash as a flux, typical of the 18^th^–19^th^ century Bohemian glass production [64, 68, 94]. Typologically and chemically consistent beads were found in North American [88, 94], European, and West African [21, 64] sites dating after the 18^th^ century.

The colour of the blue beads is given by cobalt (233–624 ppm), which can be correlated to the presence of arsenic (928–3875 ppm), nickel (60–508 ppm), bismuth (50–576 ppm), and silver (0.3–3.6 ppm), as well as uranium in type 33 (S1 Fig). This is in line with the composition of the minerals extracted from the Erzgebirge mountain range, in Germany, one of the main sources of cobalt pigments for European glass and glaze production from the 14^th^ century onwards, and especially between the 17^th^ and 19^th^ century [99, 108, 109]. Other Europeans ores showing similar mineral associations (e.g. France, Sweden, and Bohemia) can’t however be excluded [110–112].

#### Lead and lead-alkali glass

**Fig 11:**
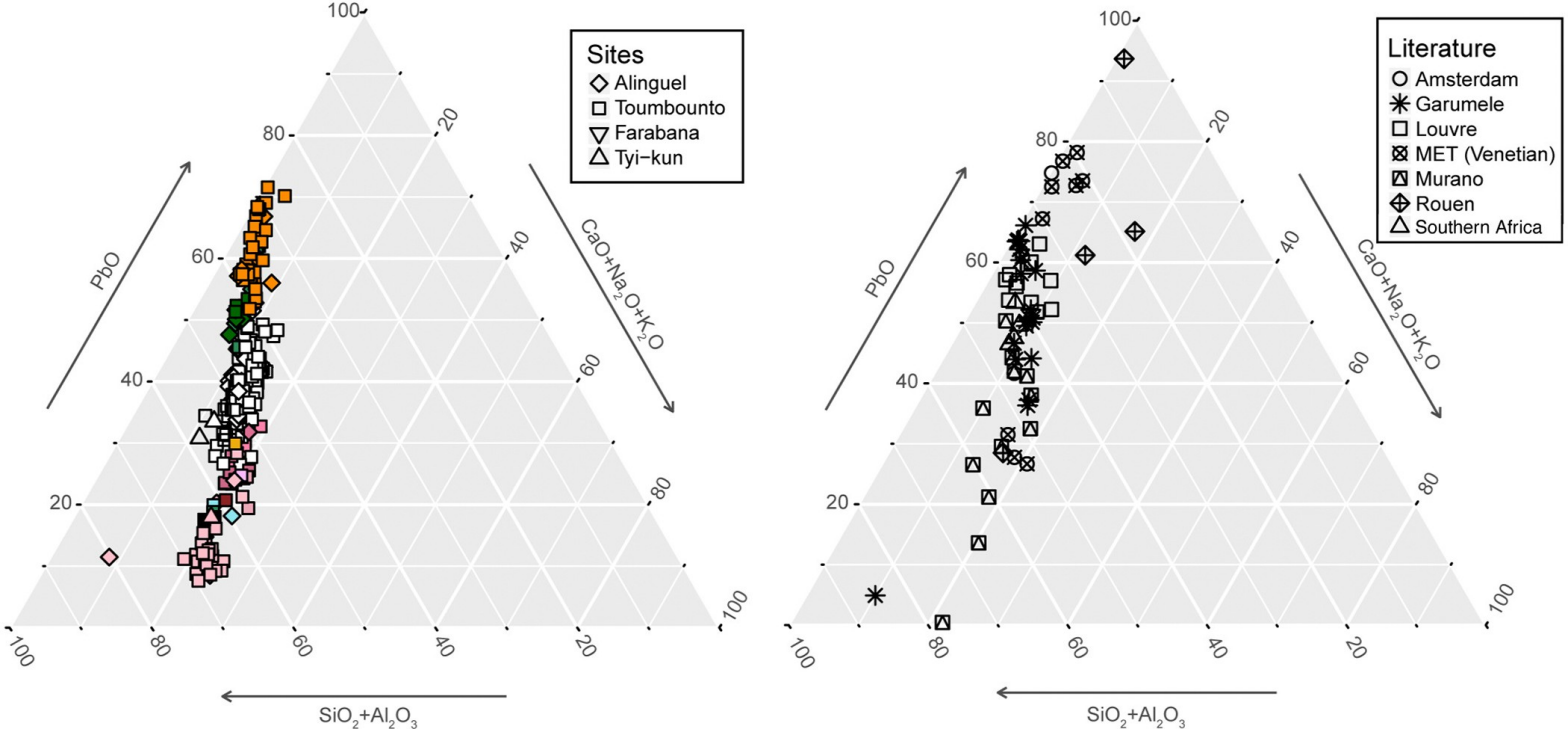
Lead and mixed-alkali lead glass composition. Comparison between lead and mixed-alkali lead glass composition and the composition of glass from Amsterdam [82], Garumele [10], Louvre [103], MET [102], Murano [75], Rouen [81], and Southern Africa [100].

The study of the potential provenance of the glass samples from this group was done by comparing the chemical composition to the Venetian glass recipe books, as well as the lead and mixed-alkali lead glass found in various European, African, and North American archaeological sites (Fig 11).

Orange and green glass show a composition similar to the glass of the same colours found in Garumele, Niger, of probable Venetian manufacture and suggested to be dated between the late 17^th^ and the 19^th^ century [10]. Moreover, the comparable Nb, Zr, and Sr content indicates that a similar source of raw materials was probably used for manufacture. Similarities can be found also with the composition of the yellow and green enamels of Venetian glass from Louvre [103], showing however higher amount of tin and antimony oxides, and slighter higher soda content. Finally, the composition of the yellow glass can be compared to the theorical composition of the Venetian *anime* that were mixed with transparent glass to create yellow opaque glass in the 19^th^ and 20^th^ century [75].

Arsenic opacified white glass is mentioned in the Venetian recipe books starting from the 18^th^ century CE. The arsenic white monochrome beads from our collection can be compared to the opaque *Girasole* and *Smalto* white glass from Murano mentioned by Moretti and Hrelich [75], as well as to the white wound beads found in Garumele [10], and to the white beads from late 18^th^–19^th^ century Sullivan’s Island site, in northern America, of probable Venetian origin [94]. On the other hand, the arsenic white glass composing our *Cornaline d’Aleppo* (as well as the ruby red glass from the same beads) has a very different composition from the red-on-white beads from Sullivan’s Island, suggested to be of Bohemian origin [94]. Correlations can be found with the composition of the red-on-white beads found in Angola, although the gold content of the ruby red glass is much lower than in our beads [99]. Although no systematic chemical analysis of red-on-white glass beads has been found in literature, the typical composition calculated from the Venetian recipe books [68, 94] is in fact comparable to our *Cornaline d’Aleppo*’s composition.

Concerning blue and turquoise lead beads, no match was found in literature. However, most of them show a very high concentration of As_2_O_3_, suggesting a production after the 18^th^ century (as for the arsenic white glass). It is also the case for the striped beads from type 50 and the blue glass composing the feather bead found in Tyi-kun (type 59). The composition of copper oxide-coloured beads from type 29 and 30 with low arsenic is on the other hand comparable with the ruby glass from *Cornaline d’Aleppo* (aside the lower content in arsenic and higher amount of copper oxide), so a Venetian production could be suggested.

#### Lead-soda silica glass of intermediate composition

This group comprises one Green Heart (TO-2-6), the three blue beads from type 24, and one of the green monochrome beads from type 34. The other samples included in this group as a result of PCA (Table 2) were included in the provenance discussion of the other groups.

Both glass composing sample TO-2-6 contain higher soda and alumina concentration compared to the other Green Hearts and the green layer show traces of chromium oxide. This suggests that the bead was produced after the 19^th^ century, when chromium green glass was produced in Venice [48]. It is also the case for the green bead from type 34 having similar base composition as the other green beads from the same type but showing a higher amount of soda and alumina and lower amount of lead, as well as a very high content of chromium oxide (0.3 wt%).

The three beads from type 24 have a soda-lead composition but unlike the other samples in the alkali-lead group previously described, they show a higher amount of soda and alumina and lower amount of potash and lime. They are coloured by cobalt and show a high amount of arsenic, placing they production probably after the 18^th^ century.

### Synthesis of exchange dynamics of glass beads in West Africa

Although it was not possible to exactly source the different glass types because of the difficulty of discerning between European production sites in the second half of the 2^nd^ millennium CE, it was possible to discern different possible supply chains for the three zones of interest, in relation to the different trade partners and time. The beads found in Old Buipe, Ghana, are comparable chemically to 17^th^ century European and North American archaeological material probably linked to the Dutch production, as well as to the tin-opacified *lattimo* glass produced between the 15^th^–17^th^ century in Venice. Glass beads are few of the rare exotic materials found in Old Buipe, which was mainly an inland commercial hub for local products between the Niger bend and the tropical forest. These glass beads were probably imported during the Dutch hegemony between the 16^th^ and the 18^th^ century. On the other hand, the much larger collection of glass beads found in the Falémé Valley, in Senegal, could be ascribed to the Venetian and the Bohemian manufacture dating between the 17^th^ and 19^th^ century, with some samples post-dating the 19^th^ century based on their chemical composition. The Falémé River Valley is well connected to the Senegalese coast through the Senegal River, hence easier to reach in the context of the Atlantic Trade. The large amount of glass beads found in various sites in the area suggests a significant demand in prestigious importation goods, that were provided by the French and English colonialists at the time. Only after the 18^th^ century the far hinterland was included in the trade system with Europe, which could justify the scarcity of glass beads found in Tyi-kun. However, it needs to be pointed out that our corpus of analysed beads from this area is too small to draw definitive conclusions.

Concerning the typology, it is interesting to notice that certain designs seem to be linked to the zone of reception, as Cornaline d’Aleppo and Green Hearts are only found in Senegal, chevron beads only in Ghana, and feather beads only in Mali. This could be linked to peculiarities in local taste or different destination of use of the items, as well as to the specific goods exchanged for the different typologies of beads.

## Conclusion

The systematic study of a large collection of glass beads excavated in five sites in Senegal, Ghana, and Mali dating between the 15th and mid-20th century led to the creation of a techno-stylistic and a chemical classification of the samples, allowing to propose hypothesis of patterns of production and trade for these items. The statistical analysis of the major, minor, and trace elements of the glass samples composing the beads, in relation with the composition of reference material from historical and archaeological collections in Europe, North America, and sub-Saharan West Africa, as well as with the techno-stylistic characteristics of the beads, allowed to identify the possible origin of the glass and to highlight differences in trade suppliers at different times. Nevertheless, more precision in sourcing the materials could be gained having access to a larger chemical database of both archaeological and historical glass, especially concerning the trace element composition, which would maybe allow to differentiate between the very similar European productions of the time.

## Supporting information

S1 Table Techno-stylistic classification of the beads. Morphological, optical, and manufacturing characteristics of each type of beads showed in Figs 2 and 3. Abbreviations: **Category:** Mon.: Monochrome, C.A.: *Cornaline d’Aleppo*, G.H.: Green Heart. **Site:** AL: Alinguel, TO: Toumbounto, OB: Old Buipe, FA: Farabana, TK: Tyi-kun. **Shape:** IR: irregular. **Colour:** w: white, cls: colourless, p: pink; r: red, g: green, b: blue, o: orange; y: yellow, blk: black. **Diaphaneity:** OP: opaque, TL: translucid, TR: transparent. The **+** sign in the colour and diaphaneity sections refers to different glasses in the same layer of the bead. The number of layers, colour, and diaphaneity of glasses in brackets refer to some of the beads of the same type. **Technique:** D: drawing, W: wounding, M: moulding. **n.d.**: indeterminate.
(PDF)

S2 Table Chemical composition of the glass samples. Full oxides (wt%) and elements (ppm) composition of the glass samples composing the monochrome and polychrome beads analysed by LA-ICP-MS.
(XLSX)

S3 Table Average base composition of each chemical group. Average composition in wt% of the samples’ base glass for each chemical group.
(XLSX)

S4 Table Average composition of each sub-group. Average concentration of the main oxides (wt%) and elements (ppm) of each chemical sub-group.
(XLSX)

S1 Fig Correlation between cobalt and other trace elements. Correlation between cobalt and the main trace elements usually found in association with in ores.
(PDF)
