# Supplementary material for: The systematic techno-stylistic and chemical study of glass beads from post-15th century West African sites
Source: PLoS One. 2025 Feb 10;20(2):e0318588. doi: 10.1371/journal.pone.0318588 (PMC11809889; doi:10.1371/journal.pone.0318588)
Supplement: S1 Table — Morphological, optical, and manufacturing characteristics of each type of beads showed in Figs 2 and 3. Abbreviations: Category: Mon.: Monochrome, C.A.: Cornaline d’Aleppo, G.H.: Green Heart. Site: AL: Alinguel, TO: Toumbounto, OB: Old Buipe, FA: Farabana, TK: Tyi-kun. Shape: IR: irregular. Colour: w: white, cls: colourless, p: pink; r: red, g: green, b: blue, o: orange; y: yellow, blk: black. Diaphaneity: OP: opaque, TL: translucid, TR: transparent. The + sign in the colour and diaphaneity sections refers to different glasses in the same layer of the bead. The number of layers, colour, and diaphaneity of glasses in brackets refer to some of the beads of the same type. Technique: D: drawing, W: wounding, M: moulding. n.d.: indeterminate. (PDF) [file pone.0318588.s001.pdf]

The systematic techno-stylistic and chemical study of glass beads from post-15th century West African sites

Miriam Truffa Giachet, Bernard Gratuze, Denis Genequand, Yao Serge Bonaventure Loukou, Éric Huysecom, Anne Mayor

Corresponding author: miriam.truffa@unige.ch, miriam.truffagiachet@gmail.com (MTG)

S1 Table. Techno-stylistic classification of the beads. Morphological, optical, and manufacturing characteristics of each type of beads showed in Figs 2 and 3. The size range given refers to the measurements of the smallest and of the largest bead of each type. The optical characteristics (colour, diaphaneity) of each glass composing polychrome beads are given from the more internal to the more external layer. The **+** sign in the colour and diaphaneity sections refers to different glasses in the same layer of the bead. The number of layers, colour, and diaphaneity of glasses in brackets refer to some of the beads of the same type. Abbreviations: **Category:** Mon.: Monochrome, C.A.: *Cornaline d'Aleppo*, G.H.: Green Heart. **Site:** AL: Alinguel, TO: Toumbounto, OB: Old Buipe, FA: Farabana, TK: Tyi-kun. **Shape:** IR: irregular. **Colour:** w: white, cls: colourless, p: pink; r: red, g: green, b: blue, o: orange; y: yellow, blk: black. **Diaphaneity:** OP: opaque, TL: translucid, TR: transparent. **Technique:** D: drawing, W: wounding, M: moulding. **n.d.**: indeterminate.

Type	Category	Total amount	Site	Length (mm)	Diameter (mm)	Longitudinal section	Cross section	Layers	Colour	Diaphaneity	Technique	Secondary modification
**1**	Mon.	323	AL, TO	0.7–9.1	1.6–6.0	Barrel, cylinder	Round, ellipse	1	w: N 9	OP	D	Heat-rounded ends
**2**	Mon.	1	TO	3.6	4.1	Cylinder	Round	1	w: N 9	OP	D	None
**3**	Mon.	165	AL, TO	1.0–7.0	1.9–9.9	Barrel, cylinder	Round, ellipse	1 (2)	w: N 9, (cls)	TL?, (TR)	D	Heat-rounded ends
**4**	Mon.	27	AL, TO	2.5–9.4	5.3–10.2	Cylinder	Round, ellipse	1 (2)	w: N 9, (cls)	TL?, (TR)	D	None
**5**	Mon.	7	AL, TO	2.2–3.8	6.1–8.4	Oblate	Round	1	w: N 9	OP	W	None
**6**	Mon.	18	TO	2.7–7.4	3.8–7.8	Barrel, oblate	Round, ellipse IR	1	w: N 9	OP?	D	n.d.
**7**	Mon.	1	OB	5.8	7.6	Barrel	Round	1	w: N 9	TL?	D	Heat-rounded
**8**	Mon.	3	OB	5.5–6.6	5.1–6.4	Barrel, round	Round	1	w: N 9	TL	D	Heat-rounded
**9**	Mon.	2	AL, TO	7.1–8.9	6.8–8.3	Barrel	Round	1	w: N 9	OP	W	None
**10**	Mon.	4	AL, TO, FA	7.8–8.8	4.5–6.0	Bicone, barrel	Square	1	w: N 9	OP	W	Heat-flattened sides
**11**	Mon.	4	AL	1.3–4.5	2.2–9.9	Round IR	Oblate	1	cls	TL	W	None
**12**	C.A.	106	AL, TO	1.1–4.3	2.7–6.2	Barrel, oblate	Round, ellipse	2	w: N 9.5,<br>p: 2.5R 3/10	OP,<br>TL	D	Heat-rounded ends
**13**	C.A.	5	TO	4.4–6.5	7.1–8.1	Barrel	Round	2	w: N9,<br>p: 5.0R 9/2	OP,<br>TL	D	Heat-rounded and cold-ground ends
**14**	C.A.	7	TO	7.3–9.5	7.8–10.2	Round	Round	1	p: 5.0R 8/4	TL	W	Heat-flattened ends

**15**	C.A.	2	TO	6.0–7.8	4.8–5.9	Bicone	Square	1	p: 2.5R 3/10	TL	W	Heat-flattened sides
**16**	C.A.	1	TO	n.d.	n.d.	Barrel	Round	2	p: 2.5R 2/6,<br>w: N 9	TL,<br>OP	W	Wound decoration on surface
**17**	C.A.	1	AL	9.9	6.1	Bicone	Triangle	1	p: 7.5R 3/12	TL	W	Heat-flattened sides
**18**	G.H.	24	AL, TO	1.5–4.3	2.3–5.1	Barrel	Round	2	g: 7.5GY 6/6,<br>r: 5.0R 5/6	TL,<br>OP	D	Heat-rounded ends
**19**	G.H.	41	AL, TO, FA	3.6–12.5	6.2–10.8	Cylinder	Round, ellipse	2, (3)	g: 2.5G 9/2,<br>r: 7.5R 3/8, (cls)	TL,<br>OP, (TR)	D	None
**20**	G.H.	23	AL, TO	4.7–8.2	7.3–10.0	Barrel	Round, ellipse	2, (3)	g: 2.5G 9/2,<br>r: 7.5R 3/8, (cls)	TL,<br>OP, (TR)	D	Heat-rounded ends
**21**	Mon.	32	TO	0.9–2.9	1.8–4.5	Barrel, cylinder	Round, ellipse	1	b: 10.0B 6/3	TL	D	Heat-rounded ends
**22**	Mon.	1	OB	1.3	2.3	Barrel	Round	3	cls,<br>b: 5.0PB 6/3, cls	TR,<br>OP, TR	D	Heat-rounded ends
**23**	Mon.	1	TO	2.3	3.5	Barrel	Round	1	b: 7.5B 6/2	OP	D	Heat-rounded ends
**24**	Mon.	3	TO	1.8–2.4	3.7–4.1	Oblate	Ellipse	1	b: 7.5B 4/8	OP	W	Heat-flattened ends
**25**	Mon.	1	TO	2.8	4.5	Barrel, oblate	Round	1	b: 7.5PB 2/5	OP	W	None
**26**	Mon.	1	OB	5.8	4.8	Barrel	Round	1	b: 5.0B 5/7	TL	D	Heat-rounded ends, *a speo*
**27**	Mon.	1	TO	3.6	7.5	Barrel	Round	1	b: 7.5BG 8/4	OP	W	None
**28**	Mon.	1	TO	4.5	4.5	Cylinder	Round	1	b: 5.0B 4/6	OP	D	Heat-rounded ends
**29**	Mon.	1	AL	6.1	6.0	Oblate	Round	1	b: 10.0B 6/3	OP	W	None
**30**	Mon.	1	TO	5.6	6.5	Barrel	Round	1	b: 7.5B 4/8	TL?	W	None
**31**	Mon.	3	AL, TO, TK	9.5	11.3	Cylinder	Polygon	1	b: 6.25PB 3/12	OP	M?	None
**32**	Mon.	1	OB	3.8	4.0	Round	Round	2	b: 5.0PB 4/8, cls	OP, TR	D?	Heat-rounded
**33**	Mon.	1	TO	17.4	19.3	Barrel	Ellipse	1	b: 2.5PB 3/8	TL	W	None
**34**	Mon.	12	AL, TO	1.4 – 2.7	1.7 – 5.8	Barrel, cylinder	Round	1	g :5.0G 5/4	OP	D	Heat-rounded ends
**35**	Mon.	4	AL, TO	1.5 – 2.2	2.9 – 3.9	Barrel, cylinder	Round, ellipse	1	g: 5.0G 6/6	TL	D	Heat-rounded and cold-ground ends
**36**	Mon.	1	AL	12.3	4.8	Cylinder	Round	1	g: 2.5G 6/4	OP	D	None
**37**	Mon.	7	AL, TO	6.1 – 10.0	6.8 – 8.3	Barrel	Round	1	g: 5.0G 5/4	OP	W	None
**38**	Mon.	1	TO	7.5	15.0	Bicone IR	Round IR	1	g: 5.0G 5/4	OP	W	Ground facets
**39**	Mon.	28	AL, TO	1.4 – 4.7	2.5 – 7.6	Barrel	Round, ellipse	1	o: 5.0YR 6/12	OP	D	Heat-rounded ends
**40**	Mon.	6	TO	2.0 – 4.2	3.0 – 4.4	Cylinder	Round, ellipse	1	o: 10.0YR 7/14	TL	D	None
**41**	Mon.	1	TO	3.7	4.9	Barrel	Round	1	y: 7.5Y 9/6	OP	M	None

**42**	Mon.	1	TO	3.0	6.3	Oblate	Round	1	o: 5.0Y 8/8	TL	W	None
**43**	Mon.	2	TO	5.5 – 6.7	8.2 – 8.4	Cylinder	Ellipse	1	o: 5.0Y 8/10	TL	D	Heat-flattened ends?
**44**	Mon.	1	AL	6.4	6.5	Barrel	Round	1	o: 10.0YR 7/8	OP	W?	None
**45**	Mon.	2	AL, TO	8.7	8.1	Barrel	Round	1	o: 10.0YR 7/8	OP	W	None
**46**	Mon.	1	TO	11.4	12.0	Round	Round	1	o: 5.0Y 8/10	TL	D	Heat-rounded
**47**	Mon.	1	TO	4.9	6.0	Barrel	Round	1	r: 8.75R 4/14	OP	M	None
**48**	Mon.	17	AL, TO	2.1 – 4.0	3.4 – 5.6	Barrel	Round	1	blk: 2.5Y 2/2	OP	D	Heat-rounded ends
**49**	Striped	2	TO	4.1 – 4.2	4.6 – 4.9	Barrel	Round	3	g: 7.5GY 6/6,<br>r: 5.0R 4/8,<br>w: N 9	TL,<br>OP,<br>OP	D	Heat-rounded ends
**50**	Striped	2	TO	1.8 – 2.0	2.4 – 3.1	Barrel	Round	2	b: 10.0B 4/6,<br>w: N 9	OP,<br>OP	D	Heat-rounded ends
**51**	Striped	1	OB	2.8	4.4	Barrel	Round	2	w: N 8,<br>r: 10.0R 3/8 +<br>g: 7.5BG 6/6	OP,<br>OP +<br>OP	D	Heat-rounded ends
**52**	Striped	1	TO	2.6	3.7	Cylinder	Round	2	o: 2.5Y 7/8,<br>r: 7.5R 3/10 +<br>g: 7.5GY 5/8	TL,<br>OP +<br>OP	D	Heat-rounded ends
**53**	Striped	1	OB	1.0	3.7	Oblate	Round	4	cls, w: N 8,<br>r: 10.0R 4/8 +<br>b: 6.25PB 3/12,<br>cls	TR, OP,<br>OP +<br>OP,<br>TR	D	Heat-rounded ends
**54**	Striped	1	OB	7.1	5.0	Cylinder	Round	3	b: 2.5PB 3/8,<br>r: 10.0R 4/8,<br>w: N 9 +<br>b: 2.5PB 3/8	TL,<br>OP,<br>OP +<br>TL	D	None
**55**	Striped	1	OB	n.d.	n.d.	Cylinder?	n.d.	3	b: 2.5PB 3/8,<br>r: 7.5R 3/8,<br>w: N 9	TL,<br>OP,<br>OP	D	n.d.
**56**	Striped	1	AL	4.6	6.8	Cylinder	Round	2	r: 10RP 8/4,<br>r: 10.0RP 7/6	OP,<br>OP	D	n.d.
**57**	Striped	1	OB	9.1	10.9	Barrel	Round	4	cls,<br>b: 7.5PB 3/8,<br>w: N 9.5	TR,<br>TL,<br>OP	D	Heat-rounded
**58**	Eye	2	TO	6.4 – 8.8	7.9 – 8.7	Barrel	Round	3	blk: 2.5Y 2/2,<br>y: 2.5Y 2/2 +<br>w: N 9,<br>r: 10.0YR 7/10	OP,<br>OP +<br>OP,<br>TL	W	None

**59**	Feather	1	TK	16.9	n.d.	Barrel	Round	3	r: 2.5R 3/10,<br>w: N 9,<br>b: 2.5PB 3/8	TL,<br>OP,<br>OP	W	None
**60**	Chevron	1	OB	14.2	20.1	Barrel	Round	6	b: 7.5BG 6/3,<br>w: N 8,<br>r: 10.0R 4/8,<br>w: N 8,<br>b: 6.25PB 3/12 +<br>g: 2.5 G3/6 +<br>r: 10.0R 4/8, cls	TR,<br>OP,<br>OP,<br>OP,<br>TL +<br>TL? +<br>OP, TR	D	Heat-rounded, cold-ground
**61**	Chevron	1	OB	8.4	11.9	Barrel	Round	6	b: 7.5BG 6/3,<br>w: N 9.5,<br>r: 10.0R 3/8,<br>w: N 9.5,<br>r: 6.25PB 3/12 +<br>b: 7.5B 6/2, cls	TR?,<br>OP,<br>OP,<br>OP,<br>OP +<br>OP, TR	D	Heat-rounded, cold-ground
**62**	Chevron	1	OB	8.0	9.7	Barrel	Round	6?	w: N 9.5,<br>b: 7.5B 6/2,<br>w: N 9.5,<br>r: 10.0R 3/8,<br>w: N 9.5,<br>b: 6.25PB 3/12	OP,<br>TL?,<br>OP,<br>OP,<br>OP,<br>TL?	D	Heat-rounded, cold-ground
**63**	Chevron	1	OB	6.2	6.5	Barrel	Round	7	cls, w: N 9.5, cls,<br>w: N 9.5,<br>r: 10.0R 3/8,<br>w: N 9.5,<br>b: 7.5PB 2/10	TR, OP, TR,<br>OP,<br>OP,<br>OP,<br>TL	D	Heat-rounded, cold-ground
