# Supplementary material for: The systematic techno-stylistic and chemical study of glass beads from post-15th century West African sites
Source: PLoS One. 2025 Feb 10;20(2):e0318588. doi: 10.1371/journal.pone.0318588 (PMC11809889; doi:10.1371/journal.pone.0318588)

The systematic techno-stylistic and chemical study of glass beads from post-15th century West African sites

Miriam Truffa Giachet, Bernard Gratuze, Denis Genequand, Yao Serge Bonaventure Loukou, Éric Huysecom, Anne Mayor
Corresponding author: miriam.truffa@unige.ch, miriam.truffagiachet@gmail.com (MTG)

S5 Figure. Correlation between cobalt and other trace elements.

Correlation between cobalt and the main trace elements usually found in association with in ores.

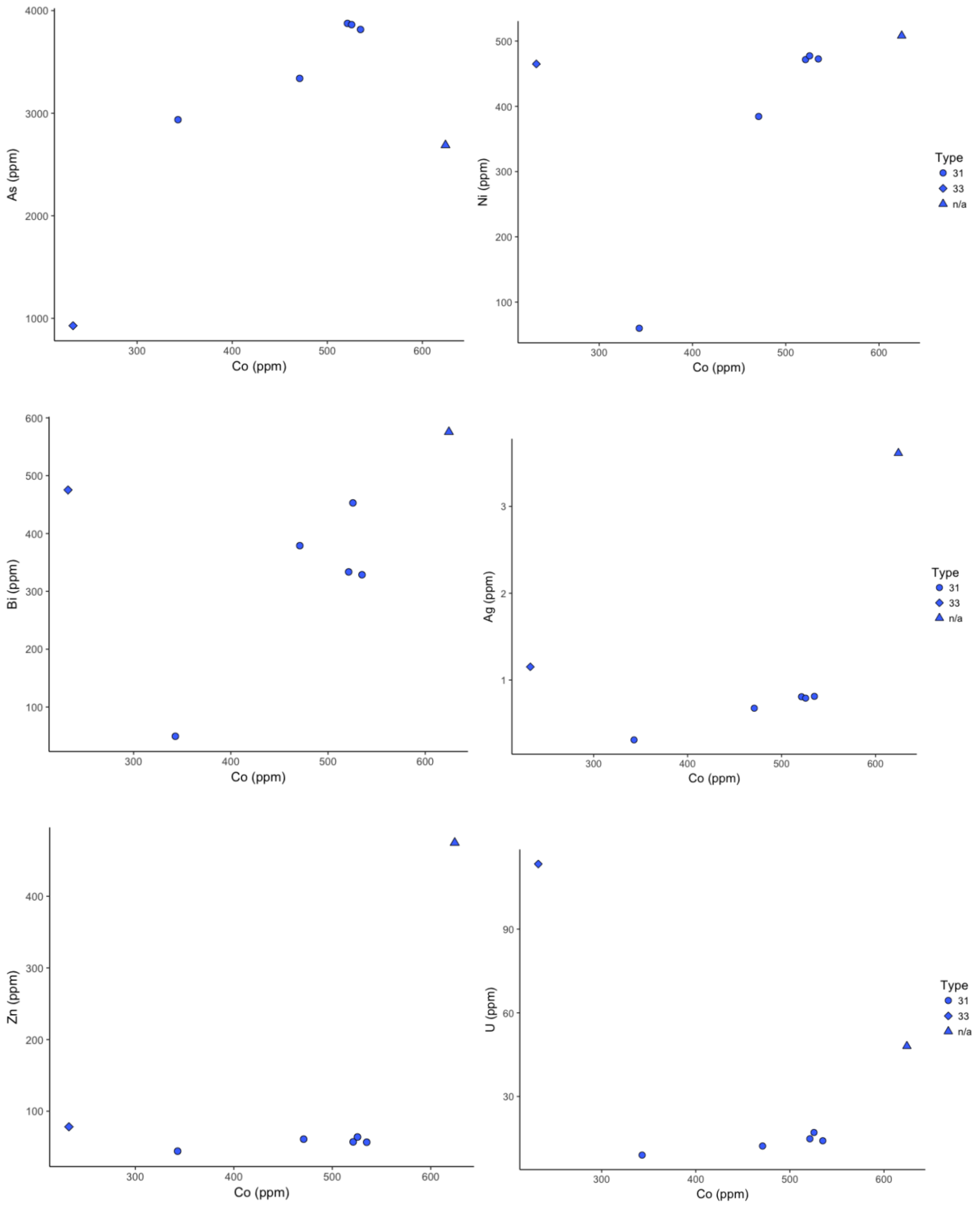

Supplement: S1 Fig — Correlation between cobalt and the main trace elements usually found in association with in ores. (PDF) [file pone.0318588.s005.pdf]
